# Silver and gold-catalyzed multicomponent reactions

**DOI:** 10.3762/bjoc.10.46

**Published:** 2014-02-26

**Authors:** Giorgio Abbiati, Elisabetta Rossi

**Affiliations:** 1 Dipartimento di Scienze Farmaceutiche, Sezione di Chimica Generale e Organica “A. Marchesini”, Università degli Studi di Milano, Via Venezian, 21 – 20133 Milano, Italy

**Keywords:** A^3^-coupling, gold, multicomponent reactions, silver

## Abstract

Silver and gold salts and complexes mainly act as soft and carbophilic Lewis acids even if their use as σ-activators has been rarely reported. Recently, transformations involving Au(I)/Au(III)-redox catalytic systems have been reported in the literature. In this review we highlight all these aspects of silver and gold-mediated processes and their application in multicomponent reactions.

## Introduction

Coinage metals (copper, silver and gold) are extensively used in the homogenous catalysis of organic reactions. Similarities and differences in the catalytic activity of these elements have been recently reviewed in an excellent book chapter by Hashmi [1]. Hashmi emphasized the difference between the “oldest” member of the family (copper), silver and the “youngest” one (gold) in terms of the literature references available for each of these three elements. Thus, the catalysis-related literature is more comprehensive for copper than for silver and gold. However, silver and gold experienced a continuous growth in interest by the scientific community. This also holds true in the field of multicomponent reactions (MCRs). A rough investigation of the literature dealing with Ag or Au-mediated MCRs published since 2000 reveals an exponential growth in the number of published papers. A deeper analysis allows discriminating between a specific class of multicomponent reactions, the A^3^-coupling reactions, which are subjected to systematic investigations, and a plethora of miscellaneous reactions. Thus, this review pursues two objectives. Firstly, we want to provide a brief overview of the most recent advances of silver and gold-mediated A^3^-coupling reactions. Seecondly, we aim for classifying the remaining classes of MCRs mediated by silver and gold species covering the literature from 2000 to early 2013. Advancements of the A^3^-coupling reactions have been recently highlighted in exhaustive and outstanding reviews by Li [2] and Van der Eycken [3], both of which cover the literature until 2010. Thus, our contribution will cover the past three years with a particular emphasis on the incorporation of the A^3^-coupling products into tandem reactions. The second goal could be achieved by classifying reactions on the basis of the involved reactants, the reaction type or the role of the catalyst.

## Review

### A^3^-coupling-type reactions

#### Silver catalysis

The catalytic direct 1,2-addition of alkynes to imines and iminium ions, generated from the condensation of amines and aldehydes, represents the most convenient method to access propargylamines [4]. Although numerous examples of the A^3^-coupling reaction have been reported, there are still many challenges and opportunities for this multicomponent coupling reaction. The expansion of its scope to include difficult substrates such as aliphatic primary amines and ammonia, the development of highly enantioselective A^3^-coupling reactions with broad substrate specificity, and the incorporation of the A^3^-coupling reaction into tandem processes are all challenges that are expected to be overcome in the near future.

**Scheme 1:**
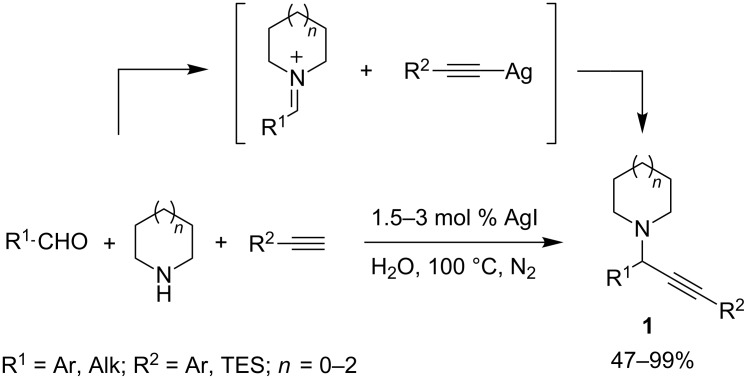
General reaction mechanism for Ag(I)-catalyzed A^3^-coupling reactions.

The first example of Ag(I)-catalyzed A^3^-coupling was reported by Li and co-workers in 2003 [5]. In this pioneering work, a simple silver(I) salt demonstrated to be able to catalyze the coupling between aliphatic/aromatic aldehydes, cyclic secondary amines and arylacetylenes in water at 100 °C under a nitrogen atmosphere. Among the different silver salts tested, AgI gave the best results. Alkyl aldehydes displayed a higher reactivity with respect to aryl aldehydes, whereas acyclic secondary amines were not well tolerated. Most importantly, the AgI-catalyzed A^3^-coupling avoided the annoying aldehyde trimerization usually observed when reacting aliphatic aldehydes under the more investigated Cu(I) and Au(I) catalysis (see below). The proposed mechanism involved the formation of a silver acetylide, which is able to react with the iminium ion generated in situ from aldehydes and amines to give the corresponding propargylamines **1** (Scheme 1).

**Scheme 2:**
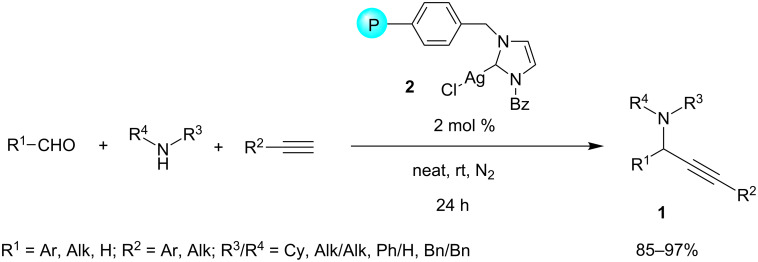
A^3^-coupling reaction catalyzed by polystyrene-supported NHC–silver halides.

As Li and Van der Eycken reported in their valuable reviews, some other silver salts (e.g., Ag_3_PW_12_O_40_ [6]_,_ AgX [7]), complexes [8], zeolites [9] and nanoparticles [10–11] have been explored to catalyze the A^3^-coupling, but only recently, silver–NHC complexes were found to be valuable catalysts for this MCR. Their first application was reported by Wang and co-workers in 2008 [12], who developed a polystyrene-supported NHC–Ag(I) complex as an efficient catalyst for the A^3^-coupling under solvent-free conditions, at room temperature, and under a nitrogen atmosphere. The in situ generated polymer-supported complexes **2** were claimed to be more active than the parent NHC–silver halides. The reactions afforded the corresponding propargylamines **1** in excellent yields starting from aromatic and aliphatic aldehydes, a wide range of secondary amines, as well as aryl and alkyl-substituted alkynes (Scheme 2). It is noteworthy that the approach tolerated challenging substrates such as formaldehyde, *o*-substituted benzaldehydes, and secondary aromatic amines. Moreover, the PS–NHC–Ag(I) catalyst was proven to be reusable at least 12 times without a significant loss of its catalytic activity. Similar PS–NHC–silver complexes were recently prepared via click-chemistry, and their aptitude to catalyze A^3^-coupling was verified [13].

**Figure 1:**
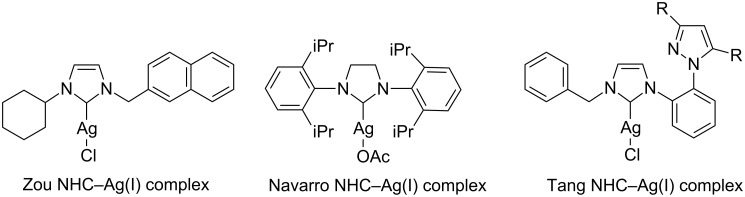
Various NHC–Ag(I) complexes used as catalysts for A^3^-coupling.

The suitability of NHC–Ag(I) complexes as catalysts for A^3^-coupling MCR was confirmed, and independently developed some years later by the research groups of Zou [14], Navarro [15] and Tang [16] (Figure 1).

**Scheme 3:**
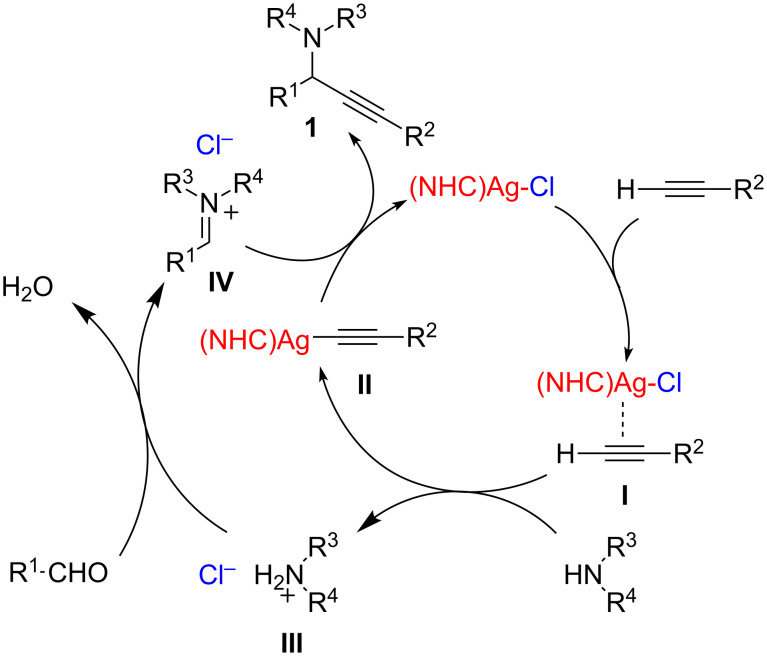
Proposed reaction mechanism for NHC–AgCl catalyzed A^3^-coupling reactions.

Zou and co-workers reported structurally well-defined *N*-heterocyclic carbene silver halides of 1-cyclohexyl-3-arylmethylimidazolylidene to be effective catalysts in a model reaction among 3-phenylpropionaldehyde, phenylacetylene and piperidine in dioxane at 100 °C in open air [14]. Although the scope was not investigated, the authors observed that the activity of the catalyst was notably affected by the nature of the anion in the order Cl > Br >> I. They argued that the true catalytic species would be a structurally stable and coordinatively unsaturated *N*-heterocyclic carbene silver halide NHC–AgX rather than a silver cation. Thus, a more detailed mechanism was proposed, in which the π-complex of the catalyst with the alkyne **I** reacts with an amine to form the silver acetylide **II** and the amine hydrohalide **III**. The latter then condenses with the aldehyde to generate the iminium halide **IV**, which reacts with the previously generated silver acetylide **II** to afford the desired product **1** and regenerate the catalyst (Scheme 3).

Bearing in mind the importance of the counter ion of the Ag complex, Navarro and co-workers developed a new saturated 1,3-bis(2,6-diisopropylphenyl)imidazolium (SIPr) silver complex, characterized by the presence of a less bulky acetoxy anion [15]. The new NHC–Ag(I) complex displayed a broad scope in A^3^-coupling reactions, tolerating alkyl and arylaldehydes (also unactivated ones), cyclic and linear secondary aliphatic amines, and terminal alkyl/aryl alkynes. It is noteworthy that the reactions occurred under mild conditions with a low catalyst loading. The solvent of choice was methanol (technical grade), but the reaction ran also well in other alcohols and acetonitrile, whereas yields were rather low in toluene.

In this context, Tang and co-workers very recently presented some original mono- and dinuclear silver–NHC complexes derived from 1-[2-(pyrazol-1-yl)phenyl]imidazole, which displayed good catalytic activity on a model A^3^-coupling reaction under Zou conditions at a slightly lower temperature (80 °C), but under an argon atmosphere [16].

**Scheme 4:**
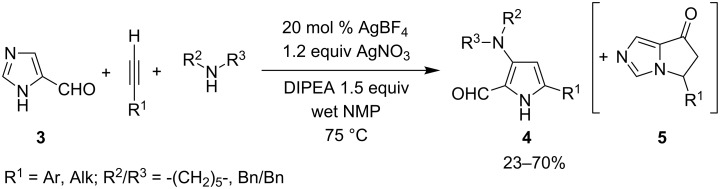
Liu’s synthesis of pyrrole-2-carboxaldehydes **4**.

An interesting Ag-promoted cascade synthesis of pyrrole-2-carboxyaldehydes involving an A^3^-coupling followed by an unusual imidazole ring opening, was reported by Liu in 2011 [17]. The authors found that propargylamines derived from the AgBF_4_-catalyzed coupling of imidazole-4-carboxyaldehydes **3**, differently substituted alkynes and secondary amines were susceptible to a subsequent in situ transformation to give 3,5-disubstituted pyrrole-2-carboxaldehydes **4** in moderate to good yields in addition to variable amounts of 5-substituted-5*H*-pyrrolo[1,2-*c*]imidazol-7(6*H*)-one **5** (Scheme 4). To obtain the best results and to reduce the formation of the pyrroloimidazolone **5**, the reactions were performed in the presence of 20 mol % of AgBF_4_, 1.2 equiv of AgNO_3_ and 1.5 equiv of DIPEA in wet NMP.

**Scheme 5:**
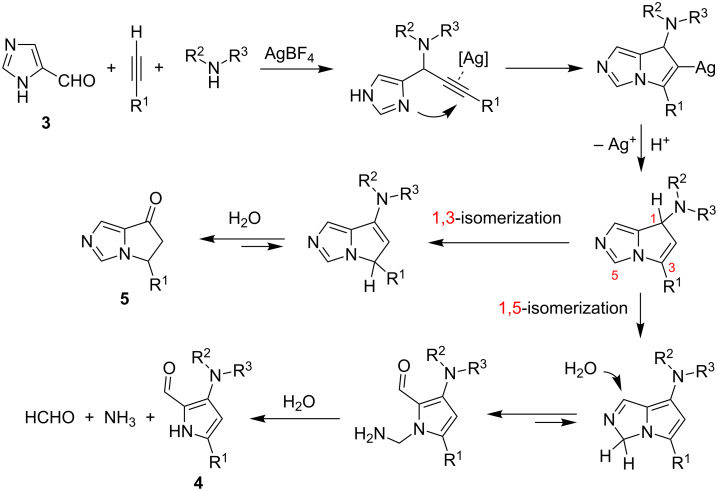
Proposed reaction mechanism for Liu’s synthesis of pyrrole-2-carboxaldehydes **4**.

Yields dramatically fall away when alkylalkynes were employed. Water was proven to be necessary in the reaction system. A series of experiments with 1-, 2- or 5-formylimidazoles and selected control reactions with the isolated propargylamine intermediate, partly in the presence of D_2_O or H_2_^18^O, were helpful to clarify the mechanism of the formation of pyrrole-2-carboxaldehydes and its byproduct. Key steps of the process are the silver-catalyzed intramolecular cyclization of propargylamine followed by a competitive 1,3- or 1,5-isomerization and a subsequent hydrolysis, yielding the pyrroloimidazolone **5** or the pyrrole **4**, respectively (Scheme 5). The 1,5-isomerization path leads to formaldehyde and ammonia, so that in the presence of silver salt the well-known silver mirror reaction could take place, thus justifying the need of at least one equiv of AgNO_3_.

A silver supramolecular complex was proposed by Sun and co-workers as an efficient catalyst for A^3^-coupling reactions between aldehydes, phenylacetylene and classical secondary amines under mild conditions (i.e., room temperature, open air, chloroform) [18]. The complex was prepared by the reaction of AgNO_3_ with 1,4-bis(4,5-dihydro-2-oxazolyl)benzene to give a tridimensional supramolecular structure characterized by three-coordinated -[Ag(NO_2_)]-L- chains, linked together by hydrogen bonds. The complex demonstrated to be more suited to aliphatic than aromatic aldehydes, whereas the presence of an EWG on the aldehyde resulted in low reaction yields.

#### Gold catalysis

**Scheme 6:**
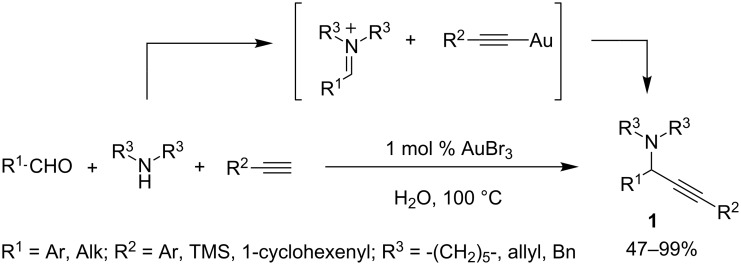
Gold-catalyzed synthesis of propargylamines **1**.

The first example of a gold-catalyzed synthesis of tertiary propargylamines from aldehydes, secondary amines and alkynes was reported by Li and co-workers [19], a bare three months before the work on silver cited above [5]. Both Au(I) and Au(III) salts demonstrated to be effective with low catalyst loading (1 mol %). Surprisingly, water was the solvent of choice, while the employment of common organic solvents gave worse results. The approach tolerated both aromatic and aliphatic alkynes and aldehydes, delivering the corresponding propargylamines **1** with fair to excellent yields. In contrast to the observations in their work on silver-catalyzed A^3^-coupling, aromatic aldehydes gave better results than aliphatic ones, and the authors ascribed this to the competitive trimerization of aliphatic aldehydes. Moreover, the approach tolerates both cyclic and acyclic aliphatic secondary amines (Scheme 6). The proposed mechanism is similar to the one suggested for the silver-catalyzed approach, involving the activation of the C–H bond of alkyne by an Au(I) species. For the AuBr_3_-catalyzed reaction, the authors argued that Au(I) could be generated in situ by a reduction of Au(III) from the alkyne.

Starting from this seminal work, many other gold catalysts, including [Au(III)salen] [20] and [Au(III)(2-phenylpyridine)Cl_2_] [21] complexes, immobilized heterogeneous catalysts [22], and gold nanoparticles (Au NP) [23–26] have been reported until 2010, as well as recognized in two recent exhaustive reviews by Li [2] and Van der Eycken [3]. In the past three years the development of new and effective nanostructured catalytic systems dominated the gold-catalyzed approach to A^3^-coupling. For example, ultrasmall gold(0) nanoparticles embedded in a mesoporous carbon nitride stabilizer [27] proved to be a highly active, selective and recyclable heterogeneous catalysts for coupling arylaldehydes, piperidine and phenylacetylene in toluene at 100 °C. One year later, the same research group obtained comparable results under identical reaction conditions by using gold(0) nanoparticles stabilized by nanocristalline magnesium oxide [28]. In this work, the scope was thoroughly investigated, and a wide range of aldehydes were tested affording the corresponding propargylamines in good to excellent yield. The method demonstrated to be suitable for challenging substrates, such as highly-activated aldehydes (i.e., nitrobenzaldehydes), whereas sterically demanding ones (i.e., *o*-substituted benzaldehydes) gave worse results. Other strengths of the approach are the ultralow catalyst loading (0.236 mol % gold) and the great TON (>400).

Periodic mesoporous organosilicas (PMOs), properly functionalized with HS/SO_3_H [29] or alkylimidazolium [30], were recently used as support for Au NP, and these heterogeneous systems were tested as recyclable catalysts in an A^3^-coupling. The former was effective in three simple model reactions as a bifunctional catalyst (Au/acid) in aqueous medium at 70 °C. The latter works well in chloroform at 60 °C and tolerates a number of substituted aryl and alkylaldehydes, cyclic secondary amines, and electron-rich arylacetylenes, affording the corresponding tertiary propargylamines in very good yields. On the basis of experiments with a reduced catalytic system and X-ray photoelectron spectroscopy (XPS) the authors suggested that Au(III) is the active component of the catalyst.

A two-step flow process catalyzed by Montmorillonite K-10 (MM K-10) and gold nanoparticles on alumina was proposed by Groß and co-workers [31] to improve the efficiency of traditional A^3^-MCRs. The flow system allows a fine-tuning of each step, i.e., ethanol as a solvent, 25 °C for aldimine formation (first step) in the MM K-10 containing packed-bed capillary reactor (PBCR), and 80 °C for the reaction with phenylacetylene (second step) in Au NP@Al_2_O_3_ containing PBCR. The system, tested with some different aryl/heteroaryl/alkylaldehydes and cyclic/acyclic secondary amines in the presence of phenylacetylene, gave the corresponding coupling products in very good to excellent yields, apart from the reactions with furfural, which obtained low yields.

An intriguing catalytic system composed of zinc oxide supported Au NP, activated by LED irradiation (plasmon mediated catalysis), was recently suggested by the group of Scaiano and González-Béjar [32] as a mild and green system to perform A^3^-MCRs. The scope was concisely explored crossing three different aldehydes (i.e., benzaldehyde, formaldehyde and 3-methylbutanal) with phenylacetylene, and three cyclic secondary amines. The coupling products were quickly obtained (2 h) at rt with yields ranging from 50 to 95%.

In the field of heterogenized gold complexes, the group of Sánchez and Iglesias [33] prepared a series of Au(I/III) complexes with some known (NHC)dioxolane and pincer-type (NHC)NN ligands, and heterogenized them on a mesoporous support, i.e., MCM-41. The authors tested them in A^3^-couplings and found that, although under homogeneous conditions the conversion to the respective propargylamine was higher than under heterogeneous ones, the heterogenized complexes were stable, recyclable for at least six cycles, active in a small amount, and under open-air conditions.

**Scheme 7:**
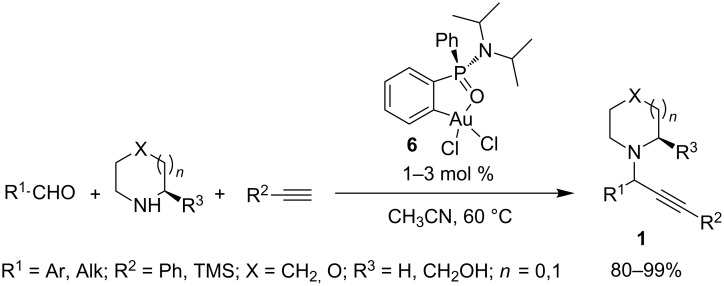
A^3^-coupling catalyzed by phosphinamidic Au(III) metallacycle **6**.

Besides the notable growing of heterogeneous catalytic systems, new gold complexes were recently developed as suitable catalysts for A^3^-MCRs under homogeneous conditions. López-Ortiz and co-workers [34] synthesized an original phosphinamidic Au(III) metallacycle **6** (via tin(IV) precursors) active at low catalyst loadings (1–3%) in acetonitrile at 60 °C under a nitrogen atmosphere. The catalyst was effective with aromatic and aliphatic aldehydes, cyclic secondary amines, and phenyl- or TMS-acetylene providing the corresponding propargylamines **1** in excellent yields (Scheme 7). When enantiomerically pure prolinol was used as amine the process took place with excellent diastereoselectivity (dr 99:1, determined by ^1^H NMR).

A series of new imidazole-based phosphane ligands were prepared by the research group of Kunz [35]. The corresponding Au(I) NP complexes displayed a potent catalytic activity in a model A^3^-coupling reaction. The best result was obtained with 0.5 mol % catalyst at 40 °C without a solvent. The scope and limitations were not investigated.

Bowden and co-workers did not propose a new catalytic system but developed a smart method to extend the lifetimes of gold(III) chloride catalysts in A^3^-MCRs by the addition of inexpensive and commercially available reagents such as CuCl_2_ and TEMPO [36]. The proposed rationale seems simple and elegant: the reduction of gold(I) (real active species) to colloidal Au(0) was responsible for the deactivation of the catalyst. CuCl_2_ was able to reoxidize Au(0) to Au(I) which increased the number of turnovers (up to 33 cycles). The Cu(I) was oxidized back to Cu(II) by TEMPO. Also O_2_ had a role in this cycle, probably as a reoxidizing agent for TEMPO.

**Scheme 8:**
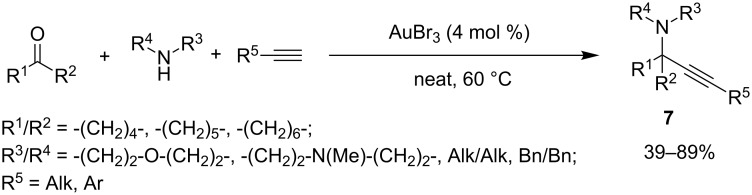
Gold-catalyzed KA^2^-coupling.

Another challenge in an A^3^-coupling strategy is its transformation in an effective KA^2^-MCR, that is, the substitution of aldehyde partners with less reactive ketones. This issue was partially solved by Ji and co-workers, who found with AuBr_3_ (4 mol %), no-solvent and 60 °C the best conditions to react alkyl ketones, secondary amines and aryl/alkylacetylenes to give the corresponding propargylamines **7** containing a quaternary carbon center [37] (Scheme 8). Aliphatic alkynes and acyclic amines gave the corresponding products in low yields, whereas the methodology was ineffective for aromatic ketones.

Gold-catalyzed A^3^-MCRs were also applied with the aim to functionalize particular molecules or were employed as a key step for the synthesis of more complex structures in domino approaches.

**Scheme 9:**
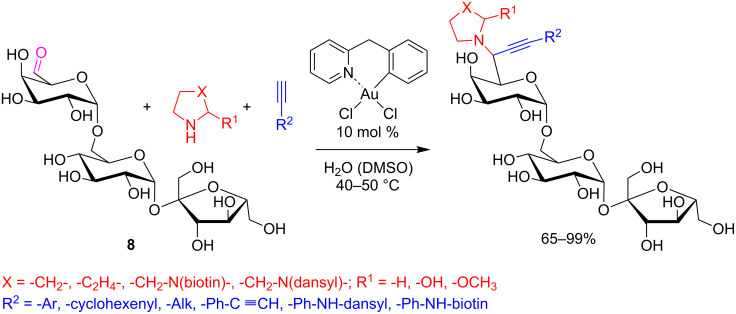
A^3^-coupling applied to aldehyde-containing oligosaccharides **8**.

For example, Che, Wong and co-workers successfully applied A^3^-coupling to aldehyde-containing oligosaccharides **8** [38]. The best catalyst for this reaction was 10 mol % of the [Au(C^N)Cl_2_] complex (HC^N = 2-benzylpyridine) in water at 40 °C. The reaction yields ranged from good to excellent, and the method allowed the introduction of alkynes and amines properly functionalized with particular groups,(i.e., dansyl and biotin), or *m*/*p*-ethynylbenzenes, suitable for further orthogonal transformation, i.e., [3 + 2] cycloaddition (Scheme 9).

**Scheme 10:**
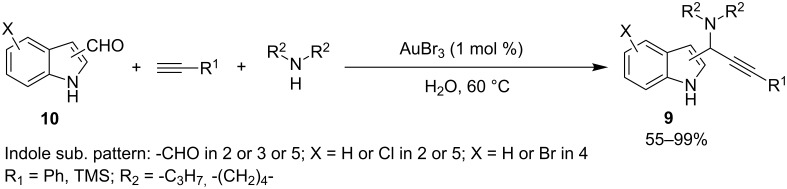
A^3^-MCR for the preparation of propargylamine-substituted indoles **9**.

Another application of an A^3^-MCR for the improvement of molecular complexity was published by Kokezu and Srinivas [39]. The authors suggested a straightforward AuBr_3_-catalyzed route to 2-, 3-, or 5-propargylamine substituted indoles **9**. The reactions were performed in water at 60 °C starting from indolecarboxaldehydes **10**, phenyl- and trimethylsilylacetylenes and cyclic/acyclic secondary amines, with the reaction yields ranging from fair to excellent (Scheme 10).

**Scheme 11:**
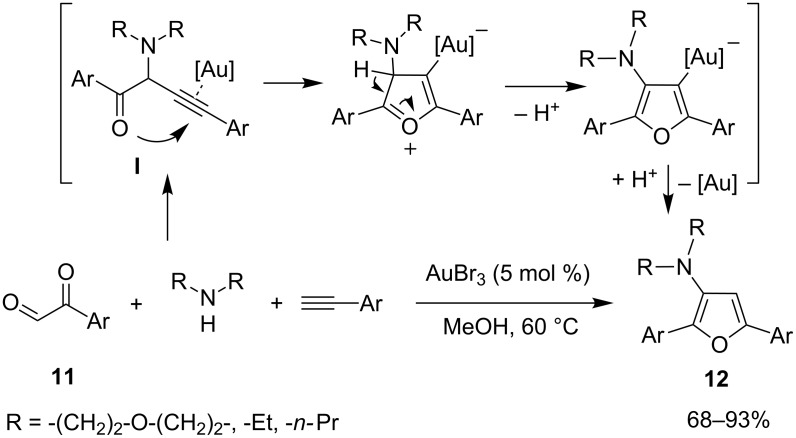
A^3^-coupling interceded synthesis of furans **12**.

Two elegant examples of cascade reactions involving an A^3^-MCR for the synthesis of valuable heterocyclic scaffolds were recently reported by the research groups of Liu and Fujii/Ohno. The Liu group developed a smart approach to furans starting from arylglyoxals **11**, secondary amines and arylacetylenes in methanol under a nitrogen atmosphere [40]. In this reaction, the best catalyst was AuBr_3_ (5 mol %) and the optimal temperature was 60 °C. The aryl moieties on alkynes and glyoxals tolerated the presence of ED and EW groups. The proposed mechanism implied the coupling among reaction partners to give an α-amino-β,γ-ynone intermediate **I** capable to undergo a 5-*endo-dig* cyclization by an intramolecular attack of the oxygen nucleophile to the Au-activated triple bond. Aromatization and protodeauration closed the catalytic cycle to give furans **12** and to regenerate the catalyst (Scheme 11).

**Scheme 12:**
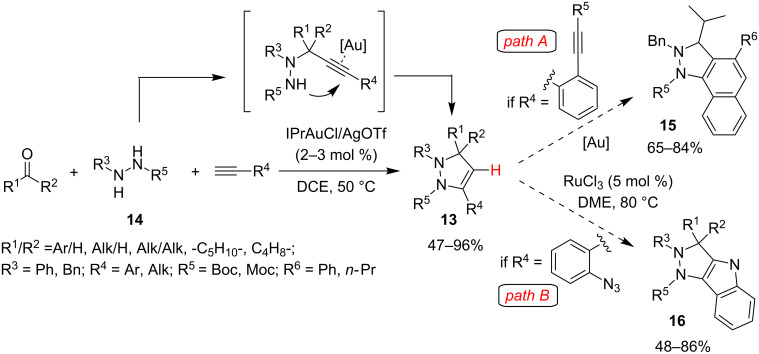
A^3^/KA^2^-coupling mediated synthesis of functionalized dihydropyrazoles **13** and polycyclic dihydropyrazoles **15** and **16**.

A conceptually similar approach – and a comparable mechanism – was proposed by Ohno and Fujii for the synthesis of functionalized dihydropyrazoles **13** starting from aryl/alkylacetylenes, aldehydes – and also more challenging ketones – and *N-*Boc*-N’*-substituted hydrazines **14** [41] (Scheme 12). Among several gold complexes tested, best results were obtained with IPrAuCl/AgOTf (2–5 mol %) in DCE (AcOH for aromatic aldehydes) at 50 °C, but also the cheaper Ph_3_PAuCl/AgOTf gave respectable results. Surprisingly, AuBr_3_ was not able to promote this cascade reaction. A special feature of this approach is that when R^4^ is a *o*-alkynylbenzene a further Au-catalyzed cascade process involving C–H activation can occur to give the corresponding tricyclic naphthalene fused pyrazoles **15** (Scheme 12, path A). Moreover, in a subsequent work, the authors applied the same strategy to obtain pyrazolo[4,3-*b*]indoles **16**, a new class of CK2 inhibitors [42]. These products were obtained starting from properly substituted dihydropyrazoles **13** in which R^4^ was an *o*-azidobenzene group by a RuCl_3_ catalyzed C–H amination (Scheme 12, path B).

**Scheme 13:**
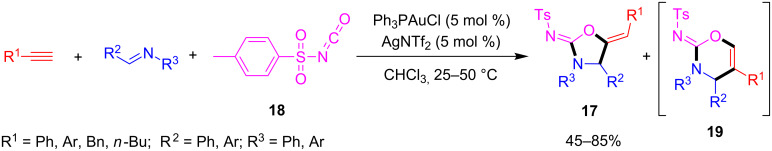
Au(I)-catalyzed entry to cyclic carbamimidates **17** via an A^3^-coupling-type approach.

As explained above, A^3^-MCR is a reaction in which the formation of a metal acetylide and its reaction with an in situ formed iminium cation are the key steps of the process. In the recent literature, there are related cascade multicomponent processes of interest, which involve gold acetylides and imines. Among them, a new Au(I)-catalyzed entry to cyclic carbamimidates **17** starting from acetylenes, imines and *p*-toluenesulfonylisocyanate (**18**) was reported by Toste and Campbell [43]. The reaction gave mainly the 5-membered carbamimidates **17** besides a variable amount of the 6-membered analog **19** (Scheme 13). The reaction partners, the more suitable catalytic system, the ratio among reagents and other reaction conditions were carefully chosen by a series of extensive experiments. In particular, the highly electrophilic *p*-toluenesulfonylisocyanate (**18**) is essential for the formation of the key intermediate. Moreover, the formation of the five-membered product **17** is thermodynamically favored by the use of small ligands in the Au complex. Only aryl substituents were well tolerated on imine and alkyne reaction partners, but imines bearing hindered *ortho* substituents or too electron-rich imines were not allowed. The reaction with alkylacetylenes( i.e., 1-hexyne), resulted in low yields and selectivity (Scheme 13).

**Scheme 14:**
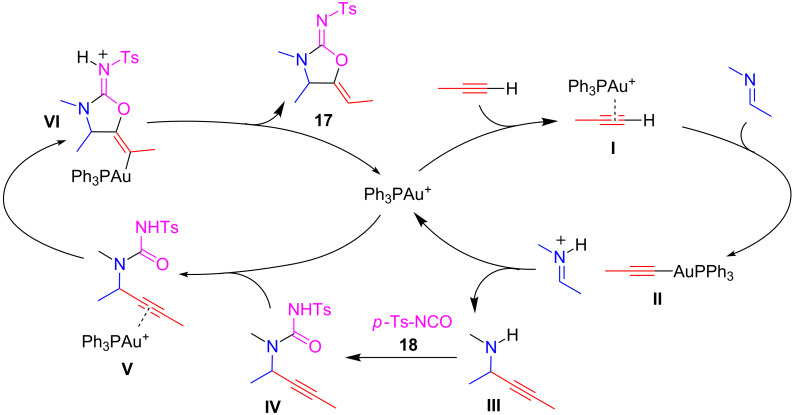
Proposed reaction mechanism for the Au(I)-catalyzed synthesis of cyclic carbamimidates **17**.

The proposed mechanism is shown in Scheme 14. The coordination of acetylene to gold produces the alkyne π-complex **I** with the acidification of the acetylenic hydrogen atom. Deprotonation by the imine produces the electrophilic iminium ion with simultaneous production of the Au(I)-acetylide **II**. An addition reaction produces propargylamine **III** and regenerates the gold cation. Amine **III** is trapped with *p*-TsNCO **18** to generate the acyclic urea **IV**, and the alkyne moiety of **IV** coordinates to gold to form a new alkyne π-complex **V**. A 5-*exo-dig* cyclization by nucleophilic attack of the urea oxygen forms the vinylgold carbamimidinium ion **VI** (the minor 6-*endo-dig* **19** product is not shown), which undergoes proton transfer to release the product **17** and regenerates the Au(I) catalyst.

**Figure 2:**
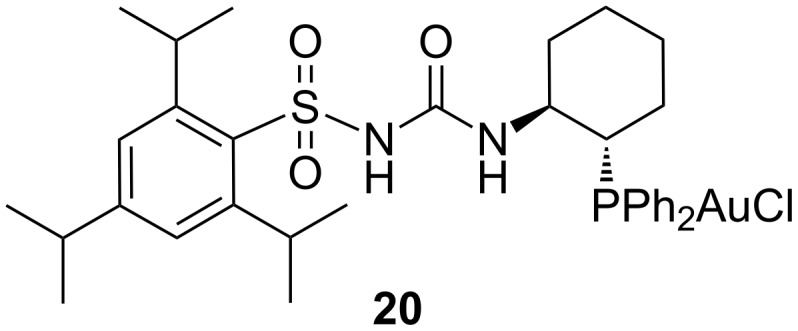
Chiral *trans*-1-diphenylphosphino-2-aminocyclohexane–Au(I) complex **20**.

The authors also developed an enantioselective version of the approach. After an in-depth preliminary screening, the catalyst and the optimal reaction conditions were found to be the original arylsulfonylurea-containing *trans*-1-diphenylphosphino-2-aminocyclohexane–Au(I) complex **20** (Figure 2), AgNTf_2_ as an additive, toluene as a solvent, rt, and a concentration of imine above 0.2 M. The obtained ee ranged from 41 to 95%.

**Scheme 15:**
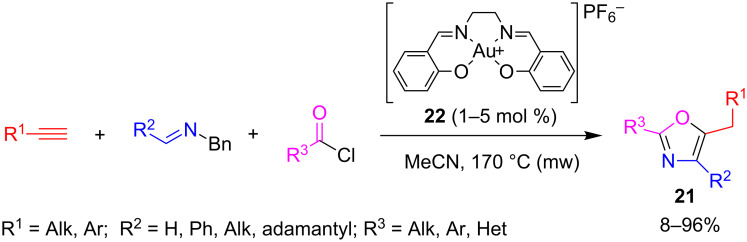
A^3^-coupling-type synthesis of oxazoles **21** catalyzed by Au(III)–salen complex.

In a similar approach, Strand and co-workers [44] worked out a new entry to oxazoles **21** starting from terminal alkynes, *N*-benzylimines and acid chlorides. The reaction was catalyzed by a Au(III)–salen complex **22** and occurred in acetonitrile at 170 °C under dielectric heating (Scheme 15).

**Scheme 16:**
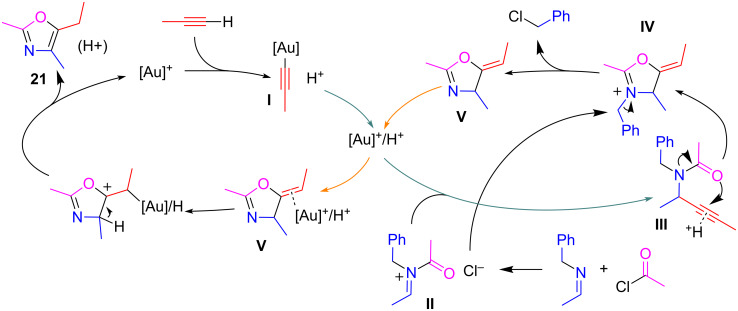
Proposed reaction mechanism for the synthesis of oxazoles **21**.

On the basis of the results of some smart kinetic experiments on ad-hoc synthetized plausible intermediates (**III** and **V**) in the presence of different amounts of catalyst (from 0 to 10 mol %) and/or 2,6-lutidine hydrochloride as a suitable proton source, the authors proposed the mechanism depicted in Scheme 16. The process involves the addition of gold-acetylide **I** to the activated *N*-acyliminium salt **II** resulting from the reaction between acyl chloride and imine, to give the propargylamide **III**. The proton released during the formation of the acetylide **I** activates the triple bond of propargylamide **III** which undergoes the attack from the amide oxygen atom. The benzyl group of the resultant iminium ion **IV** is lost as benzyl chloride by reaction with the chloride ion released during the initial imine acylation. Finally, a combination between Brønsted acid and metal catalysis, promote the isomerization of **V** to oxazole **21**. It is noteworthy, that the gold catalyst seemed to be essential only for the formation of the gold-acetylide intermediate **I**.

**Scheme 17:**
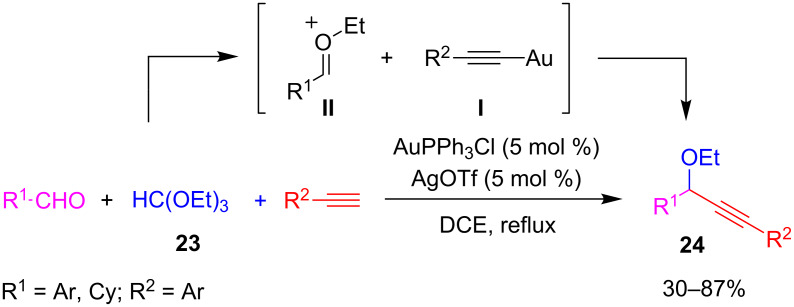
Synthesis of propargyl ethyl ethers **24** by an A^3^-coupling-type reaction.

In a different approach strictly related to Au-catalyzed A^3^-coupling, Wang and co-workers substituted the classical amine partner with triethyl orthoformate (**23**) to give the corresponding propargyl ethyl ethers **24** [45] (Scheme 17). After a brief screening for the best reaction conditions (i.e., AuPPh_3_Cl/AgOTf (5 mol %), DCE heated under reflux), the scope was investigated and best results were obtained when the reaction partners were substituted with aryl groups. In particular, the reaction of cyclohexanecarbaldehyde resulted in fair yield whereas *p*-nitrobenzaldehyde and pyridinecarbaldehyde did not react at all. During their investigations, the authors observed that AuPPh_3_/AgOTf was able to catalyze the reaction of benzaldehyde with triethyl orthoformate (**23**) to give the corresponding aldehyde diethylacetal. Consequently, the proposed mechanism involves the addition of the gold acetylide **I** to the C=O bond of an oxocarbenium intermediate **II**, formed by a Au-catalyzed reaction between aldehydes and orthoformate **23**.

### Other multicomponent processes

#### Silver assisted multicomponent reactions

Newly reported and notable synthetic strategies based on silver-mediated processes are discussed in this chapter. Silver-mediated MCRs mainly take advantage by the well-known π- and σ-philic properties of Ag(I) salts and complexes [46–48]. Thus, coordination and activation of both carbon–carbon multiple bonds or heteroatoms fulfill a MC process involving more than one chemical transformation or reaction mechanism. This part of the review is divided in sections related to the nature of the activated functionalities.

**Scheme 18:**
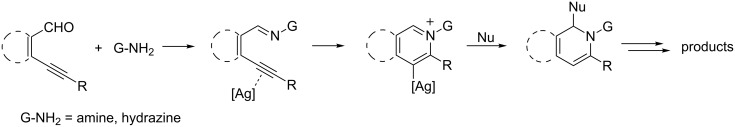
General mechanism of Ag(I)-catalyzed MCRs of 2-alkynylbenzaldehydes, amines and nucleophiles.

**Reaction involving activation of carbon–carbon multiple bonds.** This section primarily discusses cycloisomerization reactions involving the addition of imines to silver-activated carbon–carbon triple bonds [49]. Imine-based MCRs have received considerable attention in recent years [50]. The increasing interest in imine-based MCRs can be attributed to the easy preparation (even in situ) of many differently substituted derivatives from commercially available aldehydes and amines. This leads to great chemical diversity of the products of MCRs. Moreover, imines can participate in MCRs as electrophilic or nucleophilic partners, azadienes, dienophiles and 1,3-dipoles. All these reactions may benefit from the presence of a Lewis acid, a Brønsted acid or a transition metal catalyst. Silver-catalyzed MCRs involving imines in cycloisomerization reactions follow the main reaction pathway shown in Scheme 18.

**Scheme 19:**
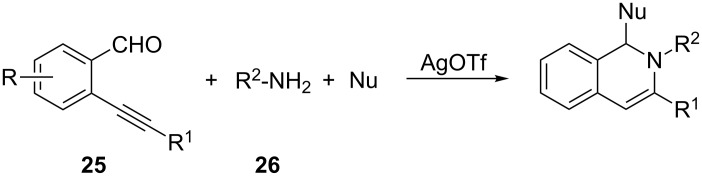
General synthetic pathway to 1,3-disubstituted-1,2-dihydroisoquinolines.

Starting from a γ-ketoalkyne [51] encompassed in a (hetero)aromatic framework, a condensation step with a suitable G–NH_2_ group (amine or hydrazine) provides the imine intermediate, which undergoes a silver-catalyzed 6-*endo-dig* cyclization, thus giving rise to a key iminium intermediate suitable to react with a third nucleophilic component (Nu in Scheme 18). In these reactions the imine acts as a nucleophile and the silver serves as a π-philic catalyst enhancing the reactivity of the triple bond toward the nucleophile. A role of the silver salt as Lewis acid in the condensation step between amine and carbonyl group has never been claimed even though it could be plausible [52]. This chemistry has been exhaustively evaluated by Wu’s group, whose main interest was the development of new MCRs as a powerful tool for the synthesis of medium-sized libraries of bioactive compounds. Thus, straightforward syntheses of 1,3-disubstituted-1,2-dihydroisoquinolines have been achieved by three-component reactions between alkynylbenzaldehydes **25** (γ-carbonylalkyne), primary amines **26** (mainly anilines) and a third nucleophilic reagent (Nu) in the presence of a silver triflate catalyst (Scheme 19).

**Scheme 20:**
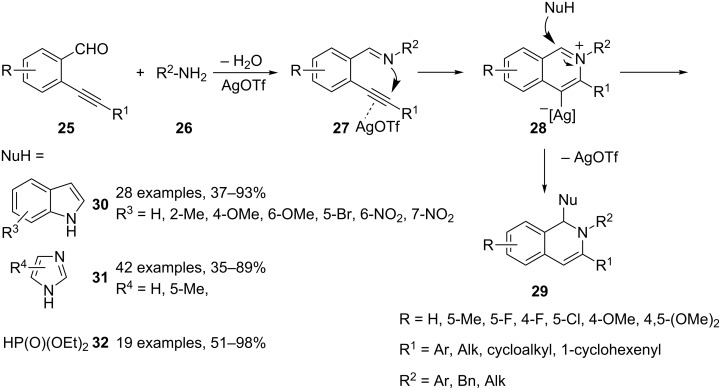
Synthesis of 1,3-disubstituted-1,2-dihydroisoquinolines **29**.

**Scheme 21:**
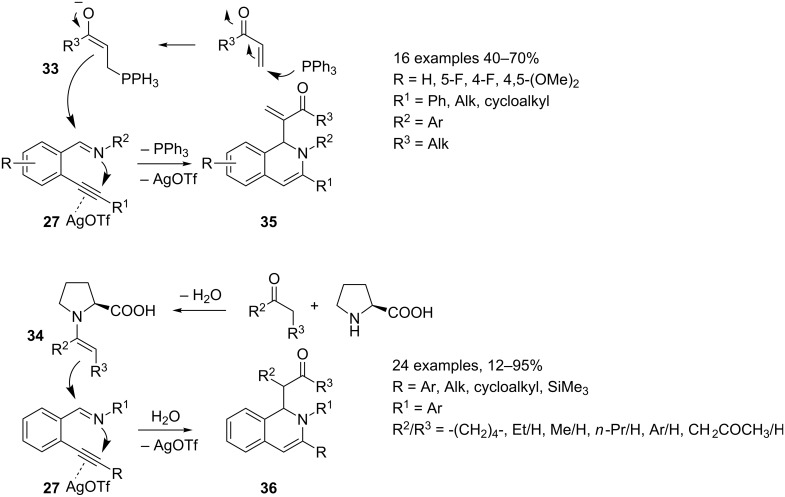
Synthesis of 1,3-disubstituted-1,2-dihydroisoquinolines **35** and **36**.

All reported reactions share the same key iminium intermediate **28** generated in situ from imine **27** via a silver triflate catalyzed 6-*endo* cyclization, but differ in the third reaction partner (Scheme 20 and Scheme 21). This can be a simple nucleophile (indole **30**, imidazole **31**, diethyl phosphite (**32**), Scheme 20) [53–55] or can be generated in situ (enolate **33**, enamine **34**, Scheme 21) [56–57] from a suitable precursor and a second catalyst (dual activation strategy) affording 1,3-disubstituted-1,2-dihydroisoquinolines **29**, **35** and **36**, respectively. The author suggested a mechanism that, starting from iminium intermediate **28**, involves the nucleophilic addition on the electrophilic carbon atom of **28** and a protodemetalation yielding the desired 1,2-dihydroquinolines **29**, **35** and **36**. The annulation step giving rise to **28**, and the nucleophilic attack on the imine C=N bond could also be synchronized. The two proposed mechanisms are described in Scheme 20 and Scheme 21, respectively.

The scope of these reactions has been examined with a wide range of substrates. Therefore, 2-alkynylbenzaldehydes can be functionalized on the aryl ring with EWG or EDG, the former better performing than the latter. However, a serious limitation on the substituents on the triple bond has been reported. Thus, phenyl and more generally aryl substituents on the triple bond are well tolerated whereas alkyl groups gave poor results. Both electron-rich and electron-poor anilines are suitable partners for these reactions, whereas alkylamines and benzylamine gave worse results. Moreover, the third partner (Nu in Scheme 19) is limited to one substrate as in the reaction employing diethyl phosphite (**32**). Indoles **30** and imidazoles **31** can bear several substituents and enolates **33** can be generated from methyl- or ethyl vinyl ketone, the corresponding α,β-unsaturated esters being unreactive. Enamines **34** arise from cyclic or linear C3–C5 ketones, acetophenones and β-diketones. It is noteworthy, that the reactions are highly regiospecific for nonsymmetric ketones. Moreover, an optical active compound could be generated during the reaction process since a chiral catalyst (proline) is used in the reactions. However, enantioselectivity was not observed by chiral HPLC analysis, and 3-pentanone gives rise to a mixture of diastereoisomers.

Following this synthetic strategy, a solution-phase parallel synthesis of 1,2-dihydroisoquinolines has been developed by Larock, providing a 105-membered library for biological assays [58]. Moreover, an extension to γ-ketoalkyne encompassed in diverse heterocyclic frameworks (quinoline, pyridine or benzo[*b*]thiophene) has been reported [59].

**Scheme 22:**
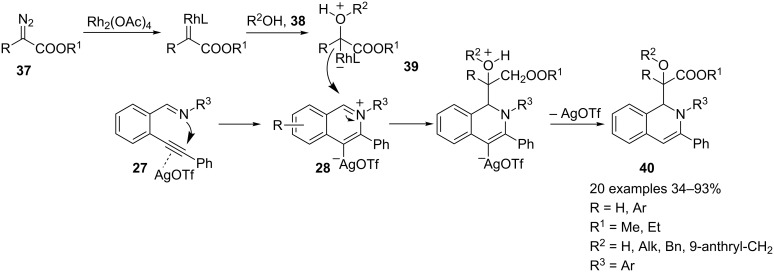
Rh(II)/Ag(I) co-catalyzed synthesis of 1,3-disubstituted-1,2-dihydroisoquinolines **40**.

Preformed 2-(1-alkynyl)arylaldimines **27** have been used in a MCR involving tandem cyclization/three-component reactions with diazo compounds **37** and water or alcohols **38** in the presence of dirhodium acetate and silver triflate cooperative catalysis resulting in excellent yields of diastereoisomeric 1,2-dihydroisoquinolines **40** (Scheme 22) [60].

Iminium intermediate **28**, generated in situ from the aldimine **27** under silver triflate catalysis is the usual electrophilic intermediate, whereas the nucleophile, in this case, is the oxonium ylide **39**. The reaction resulted in the synthesis of highly substituted 1,2-dihydroisoquinolines **40** characterized by the presence of an α-hydroxy/alkoxy-α-carboxylate carbon pendant. The oxonium ylide **39** was prepared by a well-known procedure involving a rhodium carbenoid intermediate, generated in situ from the corresponding diazoacetate **37** under Rh_2_(OAc)_4_ catalysis, and water or alcohols **38**. The scope of the reaction was thoroughly investigated. Thus, methyl aryl diazoacetates and *N*-aryl aldimines, with electronically diverse *metha* or *para*-substituents on the aryl moieties, as well as ethyl 2-diazobutanoate gave good results, only nitro and *ortho*-substituted aryl derivatives were unreactive. Interestingly, two stereocenters are generated during the reactions. However, the observed diastereoselectivities were poor, ranging from 50:50 to 76:24.

MCRs yielding isoquinoline cores are well documented in the literature and several examples involving alkynylbenzaldehydes and G–NH_2_ groups under palladium/copper [61–62], copper [63–64], copper/magnesium [65], or base [66] catalysis have been reported.

**Scheme 23:**
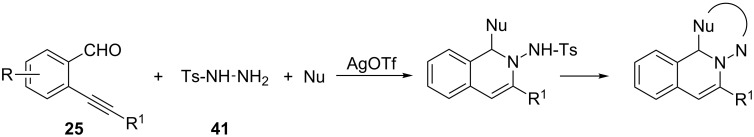
General synthetic pathway to 2-amino-1,2-dihydroquinolines.

When tosylhydrazide (**41**) is used as G–NH_2_ component, the silver promoted MCR can afford 2-amino-1,3-disubstituted-1,2-dihydroquinolines and, when the third component (Nu) bears the appropriate substituents, to polycyclic derivatives (Scheme 23).

**Scheme 24:**
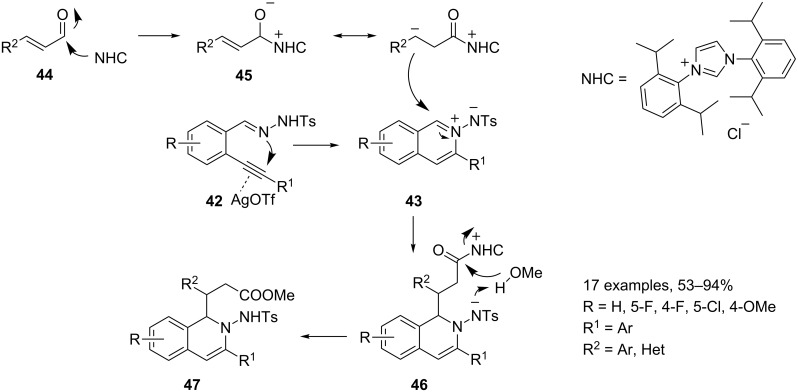
Synthesis of 2-amino-1,2-dihydroquinolines **47**.

The reactions between 2-alkynylbenzaldehydes **25** and tosylhydrazide (**41**) afford the corresponding hydrazono derivatives **42**, which, in turn, yield the isoquinolinium-2-ylamides **43** under silver triflate catalysis (Scheme 24). These new key intermediates encompass the structural motif C=N^+^–N^−^, a very useful framework for further functionalizations. Wu and co-workers widely used preformed *N*’-(2-alkynylbenzylidene)hydrazides **42** in two component reactions involving **43** as an intermediate for the construction of N-heterocycles. Moreover, in the field of MCRs, in 2010 the same authors assembled a small library of 2-amino-1,2-dihydroisoquinolines **47** starting from *N*’-(2-alkynylbenzylidene)hydrazides **42**, methanol and α,β-unsaturated aldehydes **44**. The three-component process is co-catalyzed by silver triflate and an *N*-heterocyclic carbene. (Scheme 24) [67].

**Scheme 25:**
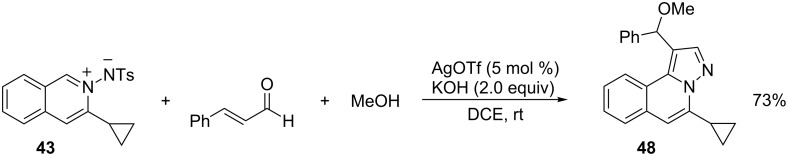
Synthesis of tricyclic *H*-pyrazolo[5,1-*a*]isoquinoline **48**.

As mentioned above, *N*’-(2-alkynylbenzylidene)hydrazide **42** could easily be transformed to isoquinolinium-2-ylamide **43** by a 6-*endo*-cyclization in the presence of silver triflate catalyst. Meanwhile, the in situ formed homoenolate **45** (derived from α,β-unsaturated aldehydes **44** in the presence of NHC catalyst, IPr) would attack the isoquinolinium-2-ylamide **43** to generate the new intermediate **46**. Subsequently, methanol would be involved in the reaction via deprotonation and the nucleophilic addition to the carbonyl group to produce the desired 2-amino-1,2-dihydroisoquinoline **47**. Concurrently, the released *N*-heterocyclic carbene would re-enter the catalytic cycle. Nevertheless, the reaction suffers from severe limitation on the nature of the R^1^ group attached to the triple bond of *N*′-(2-alkynylbenzylidene)hydrazides **42** and only aryl groups proofed to be effective. Broadening the scope of this transformation, the same authors reinvestigated the reaction on the preformed isoquinolinium-2-ylamide **43** (R = H, R^1^ = cyclopropyl) with cinnamaldehyde and methanol under silver triflate catalysis [68]. The avoidance of the use of IPr and the usage of 2 equiv of potassium hydroxide leads to a different reaction mechanism and allows for the synthesis of tricyclic *H*-pyrazolo[5,1-*a*]isoquinoline **48** (Scheme 25).

**Scheme 26:**
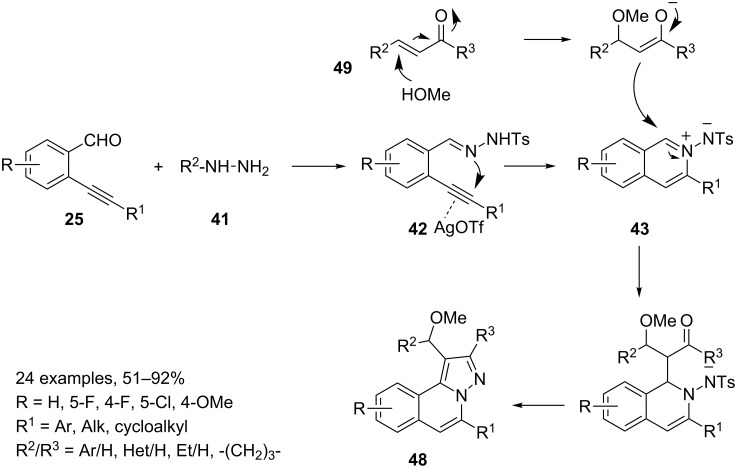
Synthesis of tricyclic *H*-pyrazolo[5,1-*a*]isoquinolines **48**.

With these results in hand, a MCR involving 2-alkynylbenzaldehydes **25**, tosylhydrazide (**41**), methanol and α,β-unsaturated aldehydes or ketones **49** was set up to synthesize a library of 24 *H*-pyrazolo[5,1-*a*]isoquinolines **48** under silver triflate catalysis (Scheme 26).

The synthesis of both 1,2-dihydroquinolines **47** and **48** takes advantage from the easy formation of the isoquinolinium-2-ylamide **43** under silver triflate catalysis. This intermediate can be trapped by other nucleophilic reagents (enamines and carbanions) or be involved in cycloaddition reactions affording tricyclic compounds by cascade processes.

**Scheme 27:**
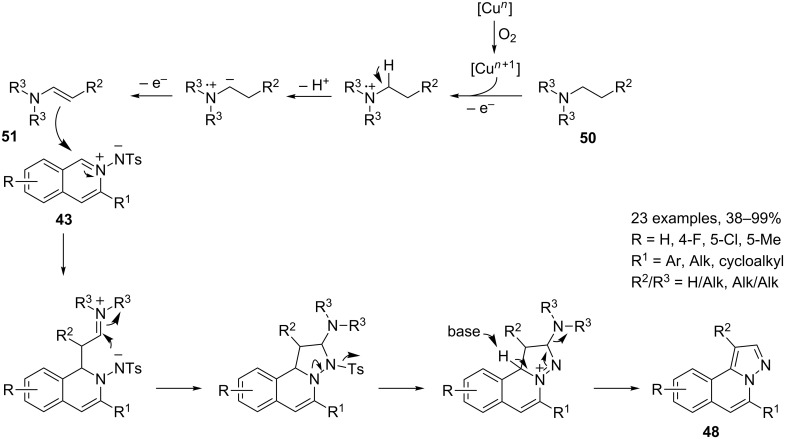
Cu(II)/Ag(I) catalyzed synthesis of *H*-pyrazolo[5,1-*a*]isoquinolines **48**.

An unprecedented, co-catalyzed reaction involving enamines **51** as nucleophilic partners, also yields the *H*-pyrazolo[5,1-*a*]isoquinoline nucleus **48**, in the presence of silver triflate and copper(II) chloride under air (Scheme 27) [69]. However, with respect to the reaction reported in Scheme 26, also affording isoquinolines of general formula **48**, a diverse arrangement of substituents can be achieved.

The proposed reaction mechanism takes into account the recent applications of an oxygen-copper catalytic system for the oxidation of aliphatic C–H bonds [70]. Thus, oxidation of the aliphatic C–H bond, alpha to the reacting amine **50**, resulted in the formation of nucleophilic enamine **51**, which is able to react with the isoquinolinium-2-ylamide **43**, thereby affording a tricyclic intermediate, which by loss of the tosyl group and base-catalyzed aromatization yields the *H*-pyrazolo[5,1-*a*]isoquinoline **48**.

**Scheme 28:**
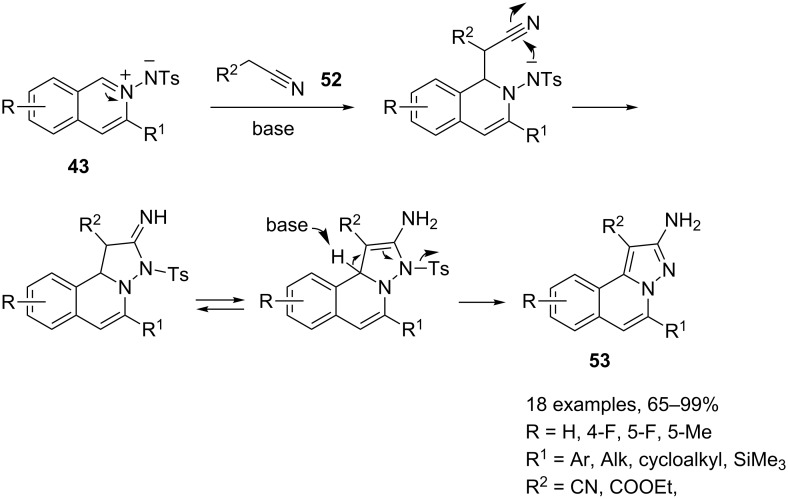
Synthesis of 2-aminopyrazolo[5,1-*a*]isoquinolines **53**.

Finally, a new series of fully aromatic pyrazolo[5,1-*a*]isoquinolines **53**, bearing an amino group in position 2 can be synthesized under silver triflate catalysis by the usual three-component reaction involving nitriles **52** as pro-nucleophiles (Scheme 28) [71].

**Scheme 29:**
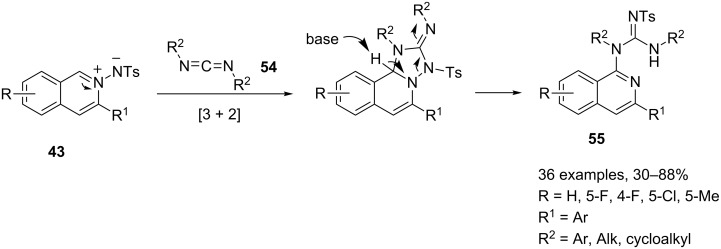
Synthesis of 1-(isoquinolin-1-yl)guanidines **55**.

Wu and co-workers successfully employed the isoquinolinium-2-ylamides **43** as an ylidic species in two-component tandem [3 + 2]-cycloaddition reactions with a series of substrates including dimethyl acetylenedicarboxylate [72], phenylacetylene [73–74], and methyl acrylate [75]. Starting from these results, a MC approach to 1-(isoquinolin-1-yl)guanidines **55** was efficiently developed by a silver triflate-catalyzed three-component reaction of 2-alkynylbenzaldehydes **25**, tosylhydrazide (**41**) and carbodiimides **54** (Scheme 29) [76].

The isoquinolinium-2-ylamide **43** undergoes a [3 + 2]-cycloaddition reaction with carbodiimide **54**. Further intramolecular rearrangement yields the desired 1-(isoquinolin-1-yl)guanidine **55**.

**Scheme 30:**
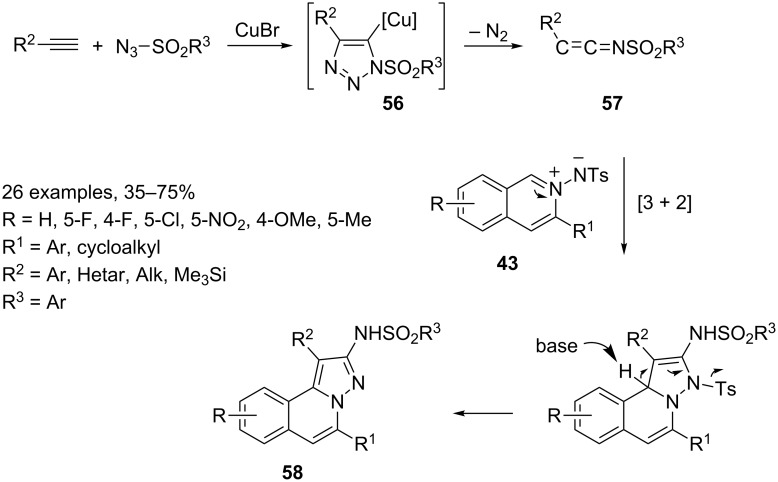
Ag(I)/Cu(I) catalyzed synthesis of 2-amino-*H*-pyrazolo[5,1-*a*]isoquinolines **58**.

**Scheme 31:**
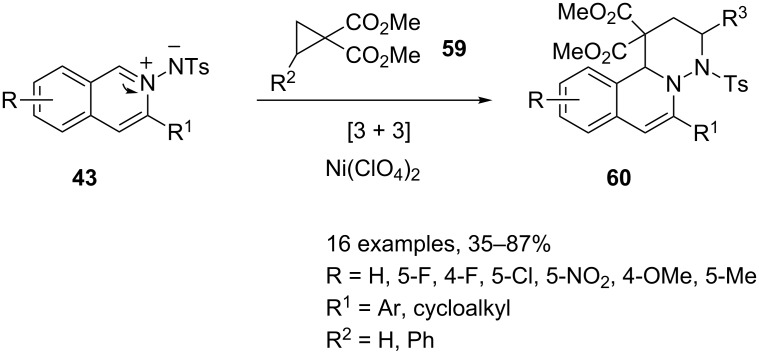
Ag(I)/Ni(II) co-catalyzed synthesis of 3,4-dihydro-1*H*-pyridazino[6,1-*a*]isoquinoline-1,1-dicarboxylate **60**.

Moreover, isoquinolinium-2-ylamides **43** can participate as 1,3-dipoles in [3 + 2]-cycloaddition reactions with in situ generated keteneimines **57** [77] or in [3 + 3] processes with dimethyl cyclopropane-1,1-dicarboxylates **59** [78]. Both these reactions are co-catalyzed, the former by silver triflate and copper bromide and the latter by silver triflate and nickel(II) perchlorate (Scheme 30 and Scheme 31).

In [3 + 2]-cycloaddition reactions between isoquinolinium-2-ylamide **43** and keteneimine **57** [79], silver salt plays the usual role of a π-philic catalyst, whereas ketene imine **57** is generated by a well-known procedure involving a copper(I)-catalyzed azide–alkyne [3 + 2] cycloaddition (CuAAC) giving rise to 5-cuprated triazole intermediate **56** which, by subsequent ring opening and loss of nitrogen gas, smoothly resulted in ketenimine **57** [80]. The overall process proceeds efficiently to generate the 2-amino-*H*-pyrazolo[5,1-*a*]isoquinolines **58** in moderate to excellent yields under mild conditions and with good substrate tolerance.

The co-catalyzed process described in Scheme 31 takes advantage of the usual formation of **43** which undergoes a [3 + 3]-cycloaddition reaction with cyclopropanes **59** under nickel perchlorate catalysis. Cycloaddition reactions of activated cyclopropanes with nitrones under Lewis acid catalysis have been previously described by Kerr and may proceed on the activated cyclopropane by a stepwise or concerted mechanism [81]. Similar mechanisms could be also operative in the reaction of ylidic species **43** for the synthesis of **60**. Good substrate tolerance and moderate to excellent yields are reported.

**Reactions involving σ-activation of carbon and heteroatoms.** This section gives an overview of the multifaceted field of silver-catalyzed processes involving a σ-activation of carbon or heteroatoms. We focus on Mannich-type reactions characterized by the addition of a nucleophile to an imine. In several MCRs with this type of reactivity, silver(I) salts and complexes have been used to activate either the nucleophile or the imine.

**Scheme 32:**
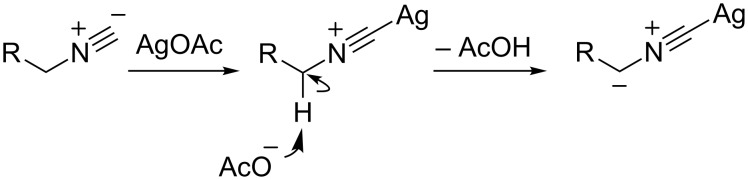
Ag(I) promoted activation of the α-carbon atom of the isocyanide group.

Isocyanides have been found to be versatile reagents in heterocyclic synthesis [82–83]. In particular, the α-metallation of isocyanides was accomplished by Schöllkopf [84] and Van Leusen [85] for the synthesis of the imidazole core structure via a Mannich-type condensation of imines. An alternative method to activate the α-carbon atom of an isocyanide group as a nucleophile is the coordination of a metal at the terminal carbon in the isocyanide group resulting in an increase in the acidity of the α-protons and thus allowing for an easy α-deprotonation with weak bases (Scheme 32) [86–87].

**Scheme 33:**
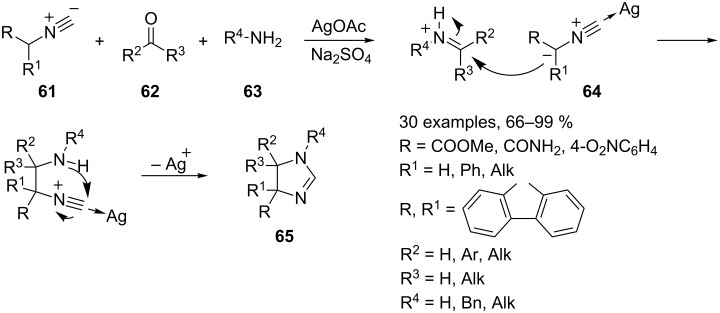
Synthesis of dihydroimidazoles **65**.

Recently, Orru’s group successfully translated the stepwise Schöllkopf–Van Leusen synthesis of dihydroimidazoles **65** in a MCR involving isocyanides **61**, aldehydes **62** and primary amines **63** [88]. Initial results, obtained in the presence of a simple dehydrating agent, were limited to the use of simple aldehydes, amines and α-acidic α,α-disubstituted isocyanides such as methyl isocyano(phenyl)acetate and 9-isocyano-9*H-*fluorene [89]. The scope of these reactions could be extended to isocyanides with other substituents by using methanol as a solvent. Further improvements can be achieved in the presence of a catalytic amount of AgOAc acting as a Lewis acid to improve the α-acidity of the isocyanide component. However, the presence of an electron withdrawing group in α-position of **61** is essential in any case (Scheme 33) [90–91].

**Scheme 34:**
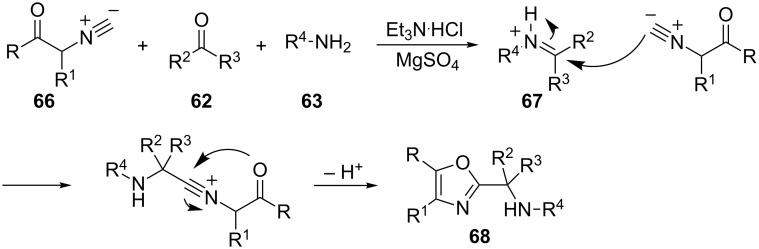
Synthesis of oxazoles **68**.

The reaction occurs via a Mannich-type addition of the deprotonated isocyanide intermediate **64** to an in situ generated iminium salt, a subsequent intramolecular cyclization and proton shift results in dihydroimidazole **65** showing predominantly *cis*-arrangment around the C4–C5 bond. However, an alternative reaction pathway, involving a concerted [3 + 2] cycloaddition of **64** to the imine, cannot be ruled out. Additionally, the use of sterically demanding amines results in lower yields. It is noteworthy, that the same reactions performed in the presence of a weak Brønsted acid instead of Ag(I) leads to oxazoles **68** when isocyano amides or isocyano esters **66** were used as substrates. The reaction proceeds through the formation of iminium ion **67** [92]. The isocyanide carbon atom is sufficiently nucleophilic to attack iminium ion **67**. Subsequent deprotonation and cyclization yields oxazoles **68** (Scheme 34).

**Scheme 35:**
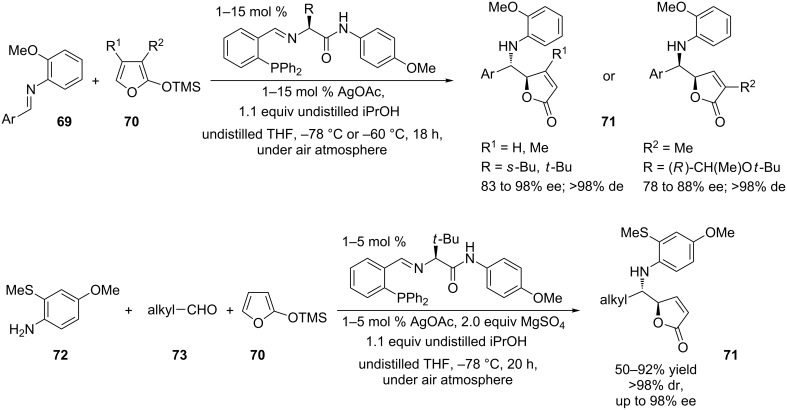
Stereoselective synthesis of chiral butenolides **71**.

Another cluster of silver-mediated Mannich-type reactions involves the enantioselective addition of siloxyfurans **70** to imines **69** (vinylogous Mannich reaction, VM) affording chiral butenolide derivatives **71** (Scheme 35). The reaction proceeds in the presence of amino acid-based chiral phosphine ligands and AgOAc via bidentate chelation of a properly substituted aldimine. Chiral phosphine–silver(I) complexes are emerging as a valuable tool for carbon–carbon bond forming reactions. These catalysts are effective in promoting enantioselective allylations, aldol reactions, Mannich-type reactions, hetero Diels–Alder reactions, 1,3-dipolar cycloadditions and nitroso aldol reactions [93]. The process was firstly accomplished with preformed aryl-substituted aldimines [94] and then developed as a MCR for less stable alkyl-substituted aldimines, which were prepared in situ from arylamines **72** and alkylaldehydes **73** to avoid decomposition [95]. Scheme 35 shows the general reaction outcome for both processes.

The two main features of the reported three-component Ag-catalyzed process are (i) the mild reaction conditions and (ii) the high degree of diastereo- and enantioselectivity. The VM process can be performed with linear, cyclic, α-branched, β-branched and *tert*-butylaldehydes as well as with heteroatom-containing aldehydes. Hence, COOMe, OBn and NHBoc substituents are well tolerated and afford the corresponding butenolide derivatives in moderate yields (44–56%). Moreover, the *N*-aryl group can be easily removed from the final compounds under oxidative conditions yielding the corresponding amino compounds.

**Scheme 36:**
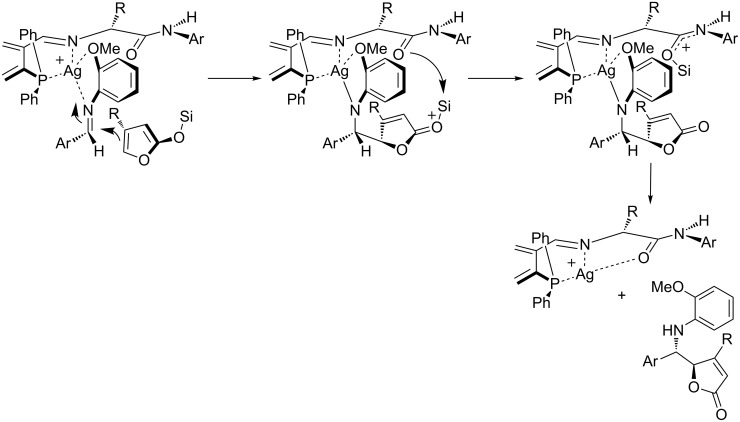
Proposed reaction mechanism for the synthesis of butenolides **71**.

An OMe substituent is essential as a directing group for aryl-substituted aldimines. Thus, the Lewis acidic chiral complex may associate with the aldimine substrate through bidentate chelation (Scheme 36). The substrate is bound *anti* to the bulky amino acid substituent (R) and reacts with the siloxyfuran via *endo*-type addition. Intramolecular silyl transfer, iPrOH mediated desilylation of the amide terminus, and protonation of the N–Ag bond delivers the final product and the catalyst. Such a pathway is not allowed for the siloxyfuran bearing a methyl group in position 3, which reacts by an *exo* addition. Alkyl-substituted aldimines can also participate in these reactions. However, they must be generated in situ (MCR). In the latter reactions, best results were obtained when arylamines **72** bear an *o*-thiomethyl and a *p*-methoxy substituent instead of a single *o*-methoxy substituent. The corresponding electron-rich aldimines are less electrophilic and subsequently more stable under the reported reaction conditions. Moreover, the authors report on a more effective association of the “softer” chelating heteroatom (sulfur) with the late transition metal, which in turn resulted in improved enantiodifferentiation via a more organized transition state.

**Scheme 37:**
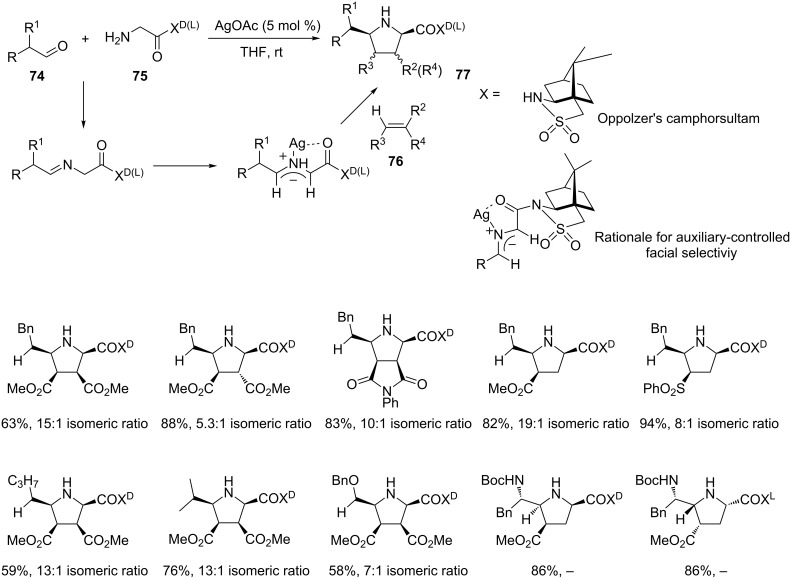
Stereoselective three-component approach to pirrolidines **77** by means of a chiral auxiliary.

Two more examples of enantioselective reactions involving silver catalysts have been recently reported. Both reactions involve amines, aldehydes and alkenes in a three-component reaction based on the cascade imine formation, azomethine ylide generation and [3 + 2] cycloaddition reaction for the synthesis of pyrrolidines. However, the adopted method to induce chirality in the final products is rather dissimilar. Thus, in 2006 Garner’s group reported the synthesis of highly functionalized pyrrolidines **77** in a MCR involving classical aliphatic aldehydes **74**, chiral glycyl sultam **75** and activated alkenes **76** (Scheme 37) [96].

The Oppolzer’s camphorsultam, incorporated in the amine **75** by means of an amide linkage, plays two different roles. On the one hand, as an electron withdrawing group, it decreases the nucleophilicity of the amine, thus avoiding the formation of detrimental Michael-type adducts with the alkene. On the other hand, it increases the α-acidity of the imine intermediate, thus favoring the azomethine ylide formation. Moreover, as a chiral auxiliary it promotes the cycloaddition governing the stereochemistry of the process. The chiral auxiliary can be removed at the end of the reaction. Another interesting peculiarity concerns the exceptionally mild reaction conditions preventing unwanted aldehyde/enol or imine/enamine tautomerization.

**Scheme 38:**
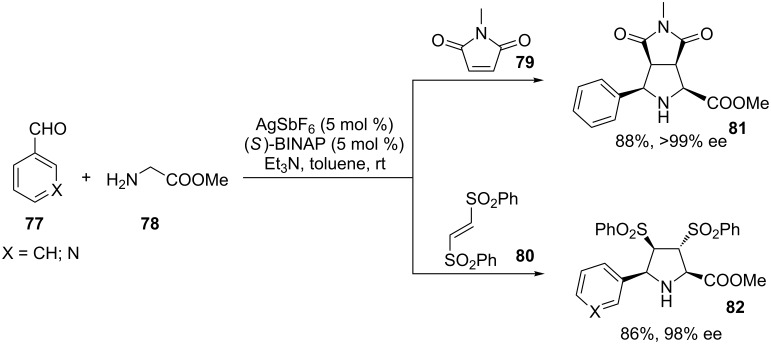
Stereoselective three-component approach to pyrrolidines **81** and **82** by means of a chiral catalyst.

Instead, an Ag(I) complex based on BINAP and AgSbF_6_ was employed as a catalyst for the enantioselective 1,3-dipolar cycloaddition reaction of azomethine ylides and alkenes for the synthesis of pyrrolidines **81** and **82** (Scheme 38) [97]. The reaction was developed mainly as a two-component reaction and only two examples of MC approaches have been included in the manuscript. The reported examples involve (hetero)aryl aldehydes **77**, methyl glycinate (**78**) and maleimide **79** or (*E*)-1,2-bis(phenylsulfonyl)ethylene (**80**) as electrophilic alkenes.

The reported work is an extension of a previous paper dealing with the use of BINAP–AgClO_4_ as a chiral catalyst in the same two-component reaction [98]. Higher enantioselectivities were rarely observed with SbF_6_^−^ being the weaker coordinating counter ion.

**Scheme 39:**
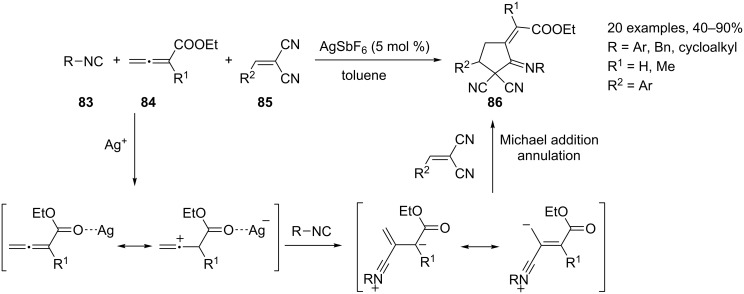
Synthesis of substituted five-membered carbocyles **86**.

An interesting application of silver catalysis in the allene chemistry field has been recently proposed by Jia and co-workers [99]. The authors got inspired by the recent development of the phosphine-catalyzed [3 + 2] cycloaddition of allenoates with electron-deficient species such as olefins and imines, which involves the in situ formation of a zwitterionic intermediate from the nucleophilic addition between allenoate and phosphine. Thus, they believed that new cycloaddition reactions could be accessed if isocyanide was employed as a nucleophile instead of phosphine. The developed reaction allows the synthesis of five-membered carbocycles **86** by the silver hexafluoroantimonate-catalyzed three-component [2 + 2 + 1] cycloaddition of allenoates **84**, dual activated olefins **85**, and isocyanides **83** (Scheme 39).

It is noteworthy, that only the external double bond of the allenic fragment is embedded in the final carbocyclic ring, whereas in the phosphine-catalyzed [3 + 2] cycloadditon process the allene moiety behaves as a traditional “three-carbon atom unit”. This behavior originates from the involvement of the isocyanide in the cyclization step.

**Reactions involving organosilver reagents.** Information about organosilver compound chemistry with respect to the coordination chemistry of silver salts and complexes is scarce in the literature. This could be related to the lower stability of these compounds, increasing in the order C_sp3_–Ag, C_sp2_–Ag, C_sp_–Ag, compared to other organometallic compounds. The majority of the screened literature discusses the use of organosilver compounds as reagents. A recent review on organosilver compounds by Pouwer and Williams exhaustively highlights all these aspects of silver chemistry [100].

**Scheme 40:**
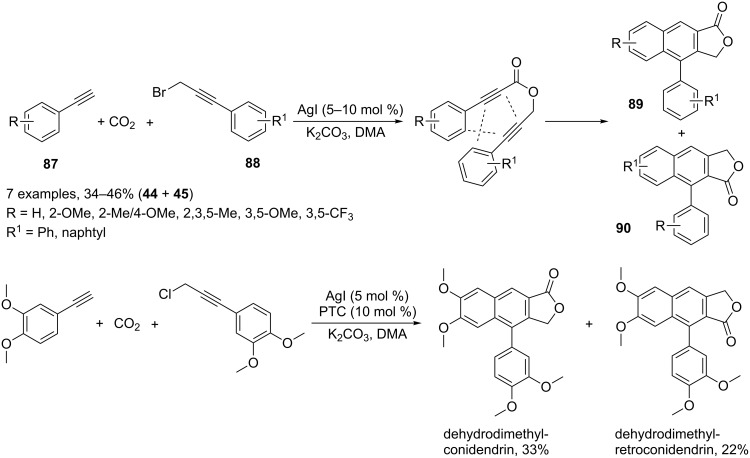
Synthesis of regioisomeric arylnaphthalene lactones.

For example, functionalized propiolic acids can be selectively prepared by an AgI catalyzed carboxylation of terminal alkynes with CO_2_ under ligand free conditions with the intermediacy of an organosilver compound, namely silver acetilide (C_sp_–Ag) [101]. The direct carboxylation of active C–H bonds of (hetero)arenes [102] and terminal alkynes [103] with CO_2_ in the presence of copper or gold-based catalysts has also been reported. However, these latter transformations require expensive ligands and often harsh bases, whereas the silver-mediated process depends on a simple but efficient catalyst such as AgI and Cs_2_CO_3_ as base. This feature has been clearly highlighted by Anastas who realized the multicomponent synthesis of regioisomeric arylnaphthalene lactones **89** and **90** from arylacetylenes **87**, carbon dioxide and 3-bromo-1-aryl-1-propynes **88** (Scheme 40) [104]. In the reaction sequence a 1,6-diyne was generated in situ and cyclized to afford the two possible regioisomeric compounds. The level of regioselectivity can be enhanced by the tuning of electronic properties of the reactant species. AgI/K_2_CO_3_ and in a greener and more efficient protocol AgI/K_2_CO_3_/18-crown-6 with 3-chloro-1-phenyl-1-propyne have been employed (Scheme 40). The latter approach was successfully adopted for the preparation of dehydrodimethylconidendrin and dehydrodimethylretroconidendrin.

#### Gold-assisted multicomponent reactions

In gold(I) and gold(III)-catalyzed reactions the metal acts as a carbophilic Lewis acid, facilitating nucleophilic addition to unsaturated systems. Moreover, also the oxophilic character of gold species has been highlighted by several authors. More recently, gold-promoted transformations involving higher oxidation states from Au(I) precatalysts have been achieved by the addition of a stoichiometric oxidant enabling two-electron redox cycles typically exhibited by other late transition metals. With respect to Ag(I)-mediated MCRs, less information can be found in the literature about the corresponding gold-mediated processes. Thus, major research efforts have been directed to the development of tandem, sequential or cascade reactions and to the area of asymmetric transformations. As reported for silver, this part of the review is divided in sections relating to the nature of the activated functionalities.

**Scheme 41:**
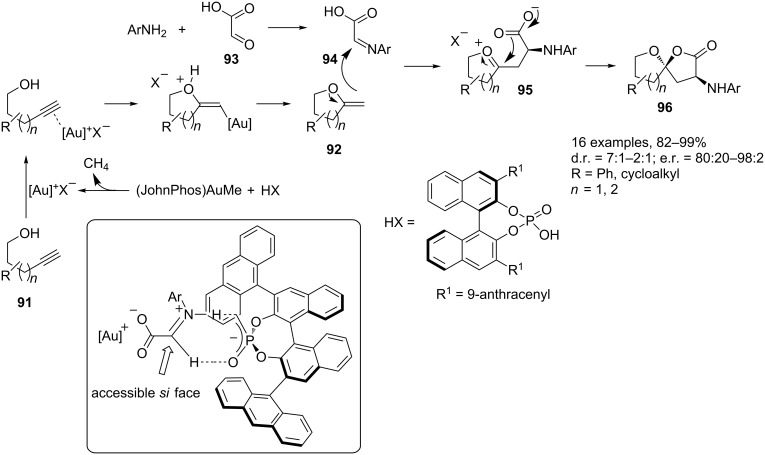
Enantioselective synthesis of spiroacetals **96** by Fañanás and Rodríguez [105].

**Scheme 42:**
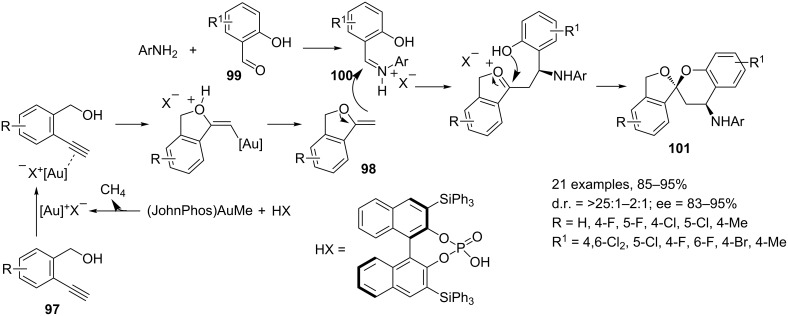
Enantioselective synthesis of spiroacetals **101** by Gong [106].

**Reactions involving the activation of carbon–carbon multiple bonds.** One of the most important reactions in gold-catalyzed synthesis is the addition of heteroatoms (O–H, N–H, C=O, C=N) to C–C triple bonds. The reactions take advantage from the high functional group tolerance and from the generally mild reaction conditions. MCRs involving this kind of reactions, however, are primarily limited to the nucleophilic addition of O–H and C=O functionalities to the Au-coordinated alkynes for the synthesis of spiroacetals, cyclic ketals and β-alkoxy ketones. The research group of Fañanás and Rodríguez [105] and the group of Gong [106] independently reported the enantioselective synthesis of spiroacetals **96** and **101** by a three-component reaction involving alkynols **91**, anilines and an α-hydroxy acid or β-hydroxyaldehydes (glyoxylic acid (**93**) or salicylaldehydes **99**), (Scheme 41 and Scheme 42, respectively). Both methodologies involve the in situ generation of a gold–phosphate complex by a reaction between (JohnPhos)AuMe and the Brønsted acid (XH) with release of a molecule of methane. These are the first examples of an intermolecular catalytic asymmetric synthesis of spiroacetals. Previously reported methodologies involved preformed substrates in intramolecular reactions [107–109].

The synthetic approach proposed by Fañanás and Rodríguez involves the coordination of the gold cation to the carbon–carbon triple bond of alkynol **91** followed by an intramolecular *exo*-addition of the hydroxy group to the alkyne which delivers the exocyclic enol ether **92** regenerating the gold-derived catalyst. The condensation reaction between glyoxylic acid (**93**) and aniline gives rise to imine **94** which, by double interaction with the gold phosphate, leads to an activated species. Subsequent nucleophilic addition of **92** to **94** gives oxonium intermediate **95**, which provides the final product **96** upon cyclization regenerating the catalyst. Interestingly, in the first catalytic cycle the main role of the catalyst is played by its cationic part, the gold(I) ion, being responsible for the activation of the alkynol **91**. Meanwhile, in the second catalytic cycle, the main role is played by the anionic part of the catalyst, the phosphate, creating the appropriate chiral environment to produce the final enantioenriched product. The model proposed for the chiral phosphoric acid catalyzed reactions between glyoxylates and enecarbamates is reported in Scheme 41 (see box). The key feature is the formation of a double hydrogen-bonded complex in which only the *si* face is fully accessible for the enol ether attack to afford the final cyclization product **96**.

As reported in Scheme 42 the method proposed by Gong and co-workers allows for the synthesis of aromatic spiroacetals **101**. The key step of the sequence is again the addition of an enol ether to an imine followed by an intramolecular cyclization reaction. The enol ether **98** is generated from *ortho*-alkynylbenzyl alcohol **97** under gold catalysis, and the imine **100** from salicylaldehyde **99** and aniline. Under the catalysis of a chiral Brønsted acid the reaction results in the synthesis of the corresponding chiral aromatic spiroacetals **101**.

**Scheme 43:**
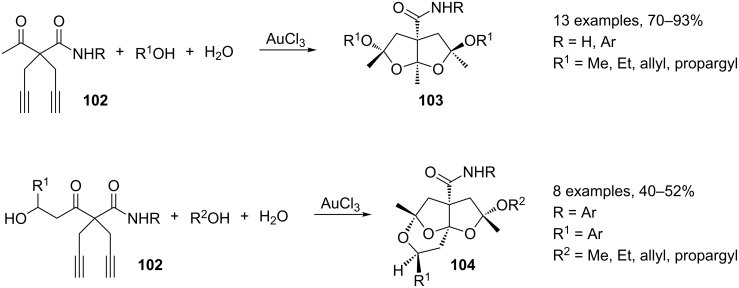
Synthesis of polyfunctionalized fused bicyclic ketals **103** and bridged tricyclic ketals **104**.

The MC synthesis of bi- and tricyclic ketals **103** and **104** takes advantage from a mechanism involving the oxyauration of a carbon–carbon triple bond [110]. Thus, starting from 4-acyl-1,6-diynes **102**, H_2_O and alkanols, under AuCl_3_-catalysis, polyfunctionalized fused bicyclic ketals **103** and bridged tricyclic ketals **104** have been prepared with a high degree of regio- and diastereocontrol. (Scheme 43).

**Scheme 44:**
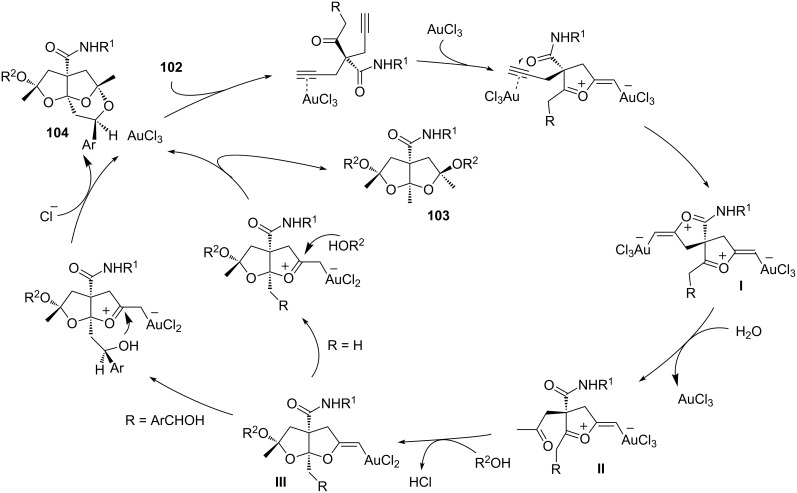
Proposed reaction mechanism for the synthesis of ketals **103** and **104**.

The reaction course can be directed toward the formation of **103** and **104** by a fine-tuning of the reaction conditions. The reactions were performed with AuCl_3_ at a catalyst loading of 3 and 5 mol %, respectively, with 1 equivalent of **102** in alkanol/water (8 mL; 25:1) (Scheme 44).

Under the optimized reaction conditions mentioned above, a double oxyauration reaction leads to intermediate **I**. The addition of water then results in the formal hydration of **I** affording dicarbonyl compound **II**. The subsequent addition of alcohol and the hydrochloric acid release affords the intermediate auric complex **III**, from which cyclic ketals **103** and **104** are formed by the inter- or intramolecular addition of alcohol, respectively. The proposed reaction mechanism also accounts for the high degree of diastereoselectivity, which can be rationalized by a series of intramolecular chiral inductions.

**Scheme 45:**
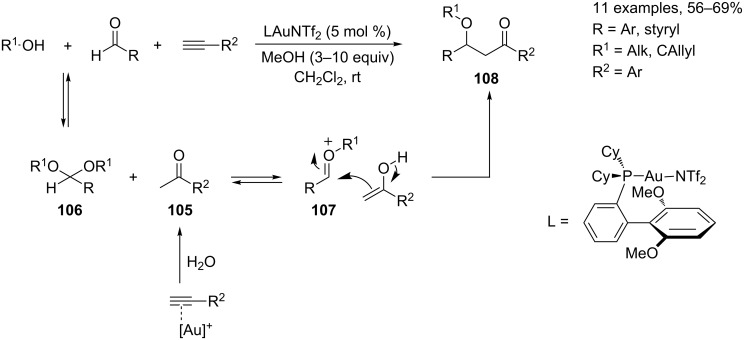
Synthesis of β-alkoxyketones **108**.

Finally, Wolfe and co-workers recently described a nice Au(I)-catalyzed MCR of readily available aldehydes, alcohols and alkynes for the synthesis of β-alkoxy ketones **108** [111]. The initial steps of the MCR encompass the Au(I)-catalyzed hydration of the alkyne to give the ketone **105** and the conversion of the aldehyde to the corresponding acetal **106**. The Au(I)-catalyzed ionization of the acetal then provides the oxocarbenium ion **107**, which is captured by the enol tautomer of ketone **105** (Scheme 45).

The authors reported a nice investigation of the involved reaction mechanism and carried out a catalytic screening devoted to the selection of the best catalytic system and optimal reaction conditions. The involvement of a protic acid (HNTf_2_) or AgNTf_2_ (used for catalyst preparation) was ruled out as control experiments performed under HNTf_2_ catalysis did not afford the β-alkoxy ketones **108**.

**Scheme 46:**
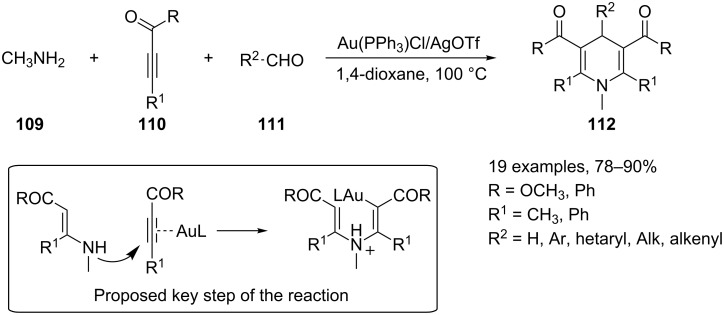
Synthesis of *N*-methyl-1,4-dihydropyridines **112**.

Newly reported examples of gold-catalyzed multicomponent reactions encompass the synthesis of nitrogen containing heterocycles, namely *N*-substituted 1,4-dihydropyridines [112] and tetrahydrocarbazoles [113]. The first example takes advantage of the ability of a cationic gold(I) catalyst to promote the formation of a new C–N bond through the hydroamination of a carbon–carbon triple bond. The three-component reaction includes methanamine (**109**), activated alkynes **110** and aldehydes **111** as reactants, a cationic gold(I) complex generated in situ from (triphenylphosphine)gold chloride and silver triflate as a catalyst, and KHCO_3_ as base. The reaction was performed in 1,4-dioxane at 100 °C and smoothly produces polysubstituted *N*-methyl-1,4-dihydropyridines **112** in good yields (Scheme 46).

The scope of the reaction was limited to the use of methanamine as a nucleophilic partner, whereas a great variety of aldehydes can be employed, aromatic, heteroaromatic, aliphatic and α,β-unsaturated aldheydes. Methyl but-2-ynoate and 1,3-diphenylprop-2-yn-1-one were tested as alkynylic counterparts. A tentative mechanistic explanation for the formation of compounds **112** was proposed by the authors. In an early stage, their theory involves a hydroamination reaction between the alkyne **110** and an enamine generated in situ by a Michael-type addition of the amine **109** on the activated carbon–carbon triple bond of a second molecule of **110** (see box in Scheme 46). The overall process closely reminds of a modified Hantzsch synthesis of dihydropyridines.

**Scheme 47:**
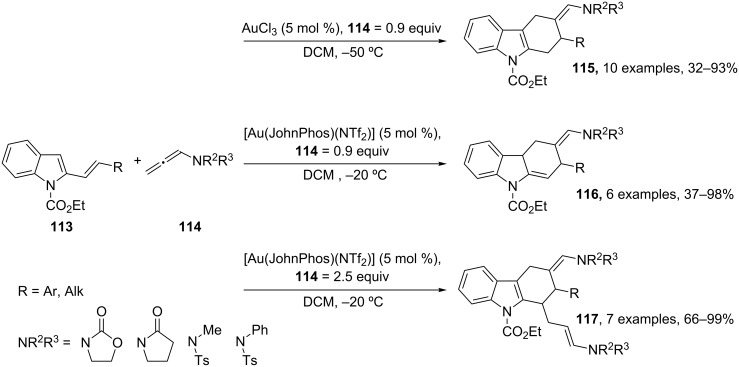
Synthesis of tetrahydrocarbazoles **115–117**.

Furthermore, among unsaturated substrates involved in gold-catalyzed MCRs, allenes could offer an incomparable versatility since they participate in [2 + 2], [4 + 2] or [4 + 3] cyclizations [114–115]. However, they have been employed in a MC process only recently [113]. A gold-catalyzed formal [4 + 2] cycloaddition of vinylindoles **113** and *N*-allenamides **114** leading to tetrahydrocarbazoles has been described. An appropriate selection of the reaction conditions enabled the selective preparation of isomeric tetrahydrocarbazoles **115** and **116** or carbazole derivatives **117** arising from an unusual gold-catalyzed multicomponent cycloaddition cascade sequence with the participation of two allene molecules (Scheme 47).

**Scheme 48:**
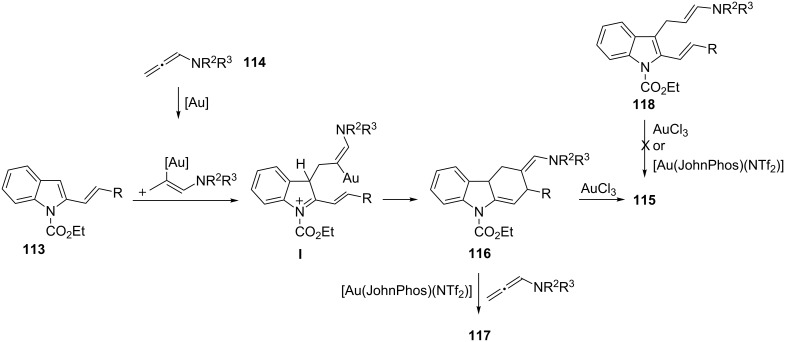
Plausible reaction mechanism for the synthesis of tetrahydrocarbazoles **115–117**.

Tetrahydrocarbazoles **115** were obtained as the only reaction products by using AuCl_3_ at −50 °C in DCM. Interestingly, a change of the catalyst to [Au(JohnPhos)(NTf_2_)] under similar reaction conditions afforded the isomeric tetrahydrocarbazoles **116** as the only diastereoisomer. As expected, the formation of multicomponent cycloadducts **117** was favored by using an excess of the allene (2.5 equiv). For this transformation, [Au(JohnPhos)(NTf_2_)] provided **117** with complete selectivity. All obtained compounds arise from a common intermediate **I** (Scheme 48). Various experiments showed that both **115** and **117** arise from compound **116**. Thus, the treatment of **116** with AuCl_3_ or [Au(PPh_3_)(NTf_2_)] led to the aromatized product **115** (>95%). In contrast, starting from **116** the use of [Au(JohnPhos)(NTf_2_)] as a catalyst in the presence of the allene (1.5 equiv) gave rise to **117** (90%), probably by a hydroarylation process. Interestingly, vinylindole **118**, independently prepared, could not be converted into **115–117** under optimized reaction conditions, pointing out that the cyclization occurred through the proposed intermediate **I**.

**Scheme 49:**
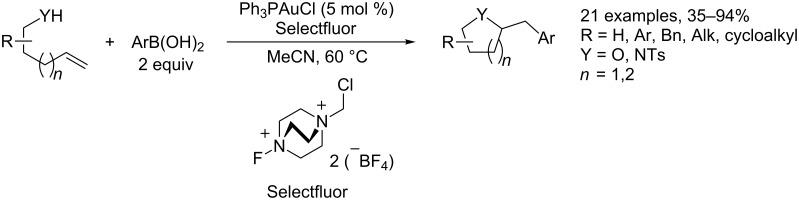
Carboamination, carboalkoxylation and carbolactonization of terminal alkenes.

**Reaction involving Au(I)/Au(III) redox cycles.** As mentioned above, transformations involving Au(I)/Au(III) redox catalytic systems have been recently reported in the literature, further increasing the diversity of gold-mediated transformation. The Au(I)/Au(III) processes can be accessed through the use of an exogenous oxidant, such as *tert*-butylhydroperoxide, PhI(OAc), or Selectfluor [116]. Inter alias, two-component Au-catalyzed heteroarylation reactions, performed in the presence of Au(I)/Au(III) redox catalytic systems, have been reported by several authors. For example, the carboamination, carboalkoxylation and carbolactonization of terminal alkenes with arylboronic acids have been implemented under oxidative gold catalysis by Zhang and co-workers (Scheme 49) [117].

**Scheme 50:**
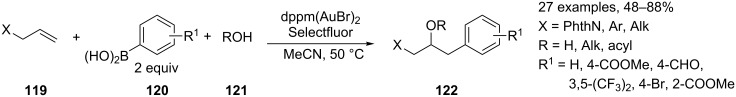
Oxyarylation of alkenes with arylboronic acids and Selectfluor as reoxidant.

The same concept has been extended to the MC heteroarylation of alkenes. Toste reported the fully intermolecular alkene heteroarylation by a gold-catalyzed three-component coupling reaction of alkenes **119**, arylboronic acids **120**, and several types of oxygen nucleophiles **121**, including alcohols, carboxylic acids, and water [118]. The reaction employs a binuclear gold(I) bromide as a catalyst and the Selectfluor reagent as the stoichiometric oxidant. Alcohols, carboxylic acids, and water can be employed as oxygen nucleophiles, thus providing an efficient entry to compounds **122** (β-aryl ethers, esters, and alcohols) from alkenes (Scheme 50).

**Scheme 51:**
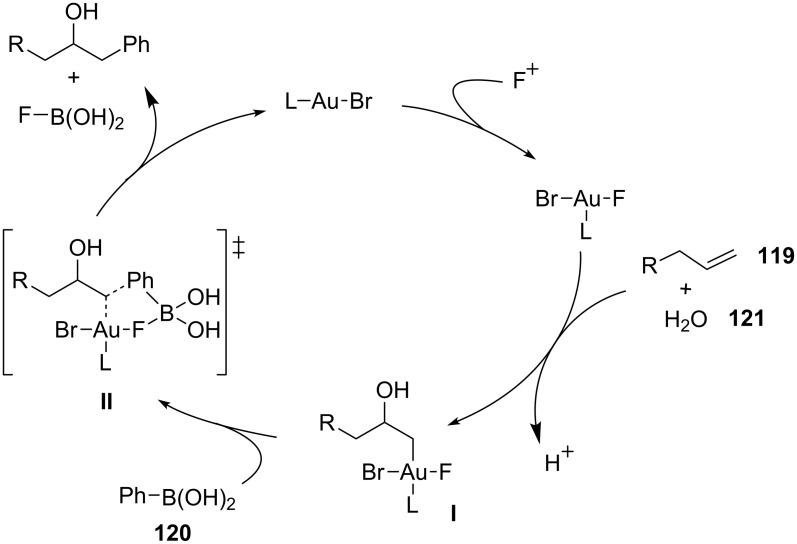
Proposed reaction mechanism for oxyarylation of alkenes.

The reactions were performed with 2 equiv of boronic acid **120** and 2 equiv of Selectfluor in MeCN:ROH (9:1) at 50 °C and in the presence of 5 mol % of dppm(AuBr)_2_ (dppm = bis(diphenylphosphanyl)methane). Ligand and halide effects play a dramatic role in the development of a mild catalytic system for the addition to alkenes. The catalyst choice is a consequence of the screening, comparing the activity of simple Ph_3_PAuX complexes and bimetallic gold complexes, accomplished by the same authors in a related two-component process [119]. The use of a bimetallic gold complexes as catalysts might minimize the formation of the unwanted bisphosphinogold(I) species [(Ph_3_P)_2_Au]^+^ observed via NMR when Ph_3_PAuCl or Ph_3_PAuBr are mixed with Selectfluor and PhB(OH)_2_. A careful investigation of the reaction mechanism resulted in the catalytic cycle reported in Scheme 51.

The first step of the catalytic cycle involves the oxidation of Au(I) into Au(III), which is the effective catalyst for the oxyauration step giving rise to the alkylgold(III) fluoride intermediate **I**. Then, the reaction of the boronic acid with intermediate **I** affords the desired final compounds with the release of fluoroboronate and the restoration of the catalyst by reductive elimination. The authors proposed a synchronized mechanism for this step, which involves the five-centered transition state **II**.

**Scheme 52:**
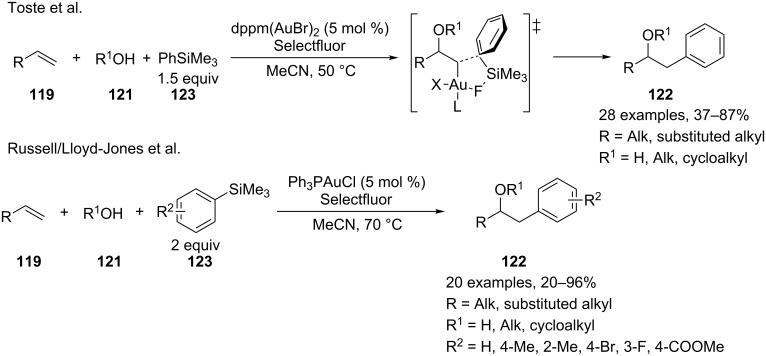
Oxyarylation of alkenes with arylsilanes and Selectfluor as reoxidant.

Moreover, Toste and Russell/Lloyd-Jones independently demonstrated that the oxyarylation of alkenes can be achieved with arylsilanes as organometallic reagents, thus avoiding the use of less benign boronic acids [120–121]. Accordingly, Toste and co-workers established that the dppm(AuBr)_2_/Selectfluor system can promote the reaction of phenyltrimethylsilane **123** with aliphatic alkenes and water or aliphatic alcohols giving rise to **122** in moderate to good yields. The Russell/Lloyd-Jones research group expanded the scope of these reactions to a series of differently substituted arylsilanes performing the reactions in the presence of commercially available Ph_3_PAuCl and Selectfluor and obtaining the desired compounds **122** with comparable yields (Scheme 52). The proposed reaction mechanism resembles the one described in Scheme 51, and the fluoride anion is probably responsible for the activation of silane without the need of a stoichiometric base. Under the reported conditions the formation of homocoupling side products of boronic acids can be reduced.

**Scheme 53:**
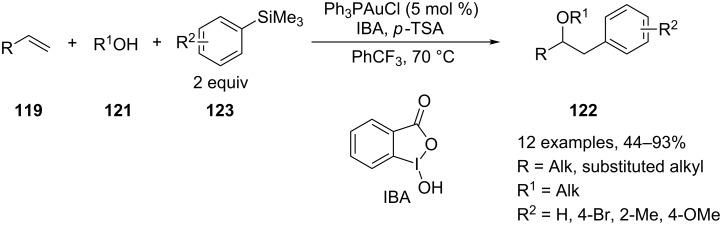
Oxyarylation of alkenes with arylsilanes and IBA as reoxidant.

More recently, Russell and Lloyd-Jones expanded the scope of these reactions to more challenging substrates such as styrenes and *gem*-disubstituted olefins, which are unreactive under the Selectfluor-based methodology reported above [122]. This goal has been achieved by introducing the 1-hydroxy-1,2-benziodoxol-3(1*H*)-one (IBA, 2 equiv) as an oxidant in addition to *p*-toluenesulfonic acid (2 equiv) as an additive and the usual gold catalysts (Ph_3_PAuCl) (Scheme 53).

The role of the acidic additive is unclear. However, the authors hinted at the in situ formation of a more electrophilic and soluble IBA-Ts oxidant. A solvent screening was carried out, and the scope of the reaction with monosubstituted, *gem*-disubstituted olefins and styrenes was carefully investigated.

## Conclusion

The development of multicomponent processes is a continuously growing research area. In this context, gold(I/III) and silver(I) are able to promote a wide range of different MCRs as both simple salts and original complexes, with a particular emphasis on the reactions involving the σ- or π-activation. These coinage metals demonstrated to be “fraternal twins” with several features in common and many peculiar differences, for example, the capability of gold to participate in a redox cycle. However, the practical and industrial importance of A^3^-coupling reactions fostered the efforts of many researchers. Other classes of silver and gold catalyzed MCRs are described and studied to a lesser extent and are often the transposition of domino reactions to multicomponent processes. Both metals ideally include all the essential features required for a catalyst devoted to control multifaceted transformations such as MCRs. Several hints could encourage the chemists’ community to mix up MCRs and silver/gold catalysis. For example, the high affinity of silver and gold catalysts for unsaturated carbon systems (e.g., alkenes, alkynes and allenes) allows performing nucleophilic additions to these systems in a chemoselective manner under exceptionally mild conditions and at the same time avoids highly reactive carbocationic intermediates. Furthermore, Au and Ag carbene intermediates, able to undergo well-defined rearrangement and/or cycloaddition reactions, are emerging as a valuable tool for the construction of carbo- and heterocyclic compounds. Au and Ag catalyzed cycloadditions itself are fields in continuous development, especially for those reactions that involve non-activated unsaturated systems. In this particular area the development of new chiral catalysts often allows to perform cycloaddition reactions in a stereocontrolled fashion. Finally, of utmost importance in the chemistry of silver and gold complexes is the possibility to control the reactivity and the properties of the metal by ligand or counterion variations. All these statements are supported by literature data and, in particular, by two topical and outstanding books, which deeply cover the chemistry of these metals [123–124].

We hope that both this review and those cited in the references could stimulate the chemists’ community toward the rationale design of new silver and gold MCRs.
